# Outbreeding and inbreeding strategies in herbaceous-shrubby communities in the Venezuelan Gran Sabana Plateau

**DOI:** 10.1093/aobpla/plz032

**Published:** 2019-06-26

**Authors:** Nelson Ramírez, Omaira Hokche

**Affiliations:** 1 Facultad de Ciencias, Instituto de Biología Experimental, Centro de Botánica Tropical, Universidad Central de Venezuela, Caracas 1041-A, Venezuela; 2 Herbario Nacional de Venezuela, Instituto Experimental Jardín Botánico Dr. Tobías Lasser, Universidad Central de Venezuela, Caracas, Venezuela

**Keywords:** Community, dichogamy, Gran Sabana Plateau, herkogamy, life form, pollination system specificity, sexual system, Venezuela

## Abstract

Breeding system, sexual system, temporal variation in sex expression and herkogamy were evaluated in seven herbaceous-shrubby communities from the Gran Sabana Plateau, Venezuela. This analysis was conducted considering the life form, substrate type, succulence, carbon metabolism, nutritional relation, successional stage, pollination system specificity and endemism of plant species. Of the 348 plant species studied, 73.8 % were hermaphrodite, 16.9 % were monoecious and 9.2 % were dioecious. Plant sexual systems such as dichogamy and herkogamy were associated with life form, nutritional relations, carbon metabolism and pollination systems. Most species were adichogamous, followed by protandrous and protogynous. Protandry was high for perennial herbs, annual herbs and trees, and protogyny was most frequent in perennial herbs. Protandrous and protogynous species were frequently anemophilous. Herkogamy was higher than non-herkogamy. Herkogamy was higher for trees, shrubs and liana; higher in monophilous and lower in anemophilous species. Most of the hermaphrodites were herkogamous and adichogamous species. In contrast, monoecy were commonly perennial herb and dichogamous species and frequently associated with anemophily. Dioecious species were trees and shrubs and with polyphilous pollination. Dioecy was the most frequent sexual system for endemic species. Hermaphrodite species were similarly distributed across plant communities. Monoecy was slightly higher for savanna and fallow than the other communities, and dioecy was higher for shrublands and secondary bushland. Most plant species were non-agamospermous, non-spontaneous self-pollinated and xenogamous. Partially self-incompatible dominated, followed by self-incompatible, partially cross-incompatible and the lowest frequency corresponded to cross-incompatible species. All these results are discussed in the context of evolutionary and ecological trends.

## Teaser

Breeding system, sexual system, dichogamy and herkogamy taking place in seven herbaceous-shrubby communities from the Gran Sabana Plateau, Venezuela, showed diverse mating strategies associated with life forms, pollination system specificity, successional stage, endemism, vegetation structure and community isolation. High levels of outbreeding strategies such as dioecy, monoecy, non-agamospermy, non-spontaneous self-pollination, xenogamy and partial self-incompatibility occurred mainly in woody species from shrublands and secondary bushland. Inbreeding strategies, non-herkogamy, spontaneous self-pollination and partial cross-incompatibility are primarily associated with herbaceous life forms from disturbed communities. These reproductive attributes are discussed in the ecological and evolutionary context.

## Introduction

Mechanisms promoting outcrossing include self-incompatibility, unisexuality, dichogamy and herkogamy (Cardoso *et al.* 2018); however, these mechanisms differ according to their effectiveness. The most successful forms for outcrossing are self-incompatibility and dioecy. Self-incompatibility is considered the most common reproduction form in neotropical plant communities (Machado *et al.* 2006), and it is frequently associated with the perennial or arborescent habit (Newstrom and Robertson 2005). On the contrary, self-compatible species are herbaceous species frequently associated with early successional seres or disturbed areas where autogamy is probably selected as a colonizing strategy and pollinator fauna is scarce and/or inefficient (Lemus-Jiménez and Ramírez 2005). Similarly, many agamospermous species are also colonizing species growing in disturbed areas (Michaels and Bazzaz 1989; Hokche and Ramírez 2008) and are well represented in neotropics (Firetti 2018).

In the case of sexuality, hermaphroditism is most common, followed by monoecy and dioecy at the community level (Machado *et al.* 2006; Tseng *et al.* 2008; and many others). Dioecious and self-incompatible are often woody species (Godin 2017 and many others) and pollination system of dioecious species is considered generalist (Matallana *et al.* 2005). In the case of monoecy, cross-pollination is promoted by preventing within-flower selfing (Willson 1983). Renner and Ricklefs (1995) and Renner (2014) found that dioecy in angiosperms seems to have evolved most frequently from monoecy, in association with wind or water pollination, and climbing growth. The evolution of monoecy may occur from hermaphroditism through gynomonoecy (Torices *et al.* 2011), and under polyphilous and wind pollination systems (Ramírez 2005*a*). In addition, dioecy and monoecy incidence at the community level may also be, in some cases, influenced phylogenetically since there are huge entirely dioecious clades of tropical woody plants; however, such effect is restricted by the vegetative dispersal and sedentary lifestyle of angiosperms (Renner 2014).

Temporal separation of sexual expression (dichogamy) and spatial separation of sexual organs (herkogamy) are widespread in outcrossing angiosperms and play a vital role in the successful functioning of blossoms (Barrett 2003; Cardoso *et al.* 2018). Through these mechanisms, pollen–stigma interference is avoided, self-fertilization is reduced and cross-pollination is promoted due to the non-simultaneous presentation of pollen and stigma (Cardoso *et al.* 2018). Survey and community studies on dichogamy and herkogamy have received little attention compared with other floral features. There are only a few studies available and those show protandry to be more common than protogyny and the occurrence of dichogamy was found lower than herkogamy (Ramírez 2005*a*). Additional studies at the community’s level examining the associations between ecological and reproductive traits contribute to understanding the ecological and the evolutionary significance of plant breeding systems under contrasting environmental conditions.

Studies addressing breeding systems, sexual systems, dichogamy and herkogamy in heterogeneous vegetation, including disturbance, situated in the same geographic areas allow fair comparisons between plant communities. In the Gran Sabana Plateau, plant species richness is high in the herbaceous-shrubby communities (Ramírez *et al.* 2012).

In these communities, epiphyte, endemic, carnivorous and parasitic plants exhibit high levels of community specialization. Life form diversity and the nutritional relations differ between plant communities. Another significant attribute is the high incidences of endemic species, which are associated with the environmental heterogeneity (Steyermark 1979; Duno de Stefano *et al.* 2009). In this context, endemism, gene flow and even community organization may be somewhat explained by breeding features. Moreover, many of plant species in these communities exhibit extended phenological periods and consequently overlapped reproductive phenology (Ramírez and Briceño 2011), which may have consequences on plant reproductive systems due to the possibility of interspecific pollination. In general, the proportion of dioecious species directly depends on the frequency of the abundance of species with endemic areas (Godin 2017) and appears to result from autochthonous speciation of dioecious lineages (Schlessman *et al.* 2014). In this context, the high level of specialization, diversity and endemism in the Gran Sabana Plateau allows evaluating the specific relationships of reproductive traits in specialized plant communities and their particular functional groups.

The primary goal in sexual traits analysis has been to assess the relative importance of various selection pressures and to understand how they interact in different situations (Thomson and Brunett 1990). The first aim of this study was to evaluate whether breeding systems, sexual systems, dichogamy and herkogamy frequencies differ among distinct herbaceous-shrubby communities in the Gran Sabana Plateau. In this context, we also evaluated the evolutionary and ecological relationships between reproductive (sexual and breeding system) and vegetative traits (life form, plant physiology and successional stage), pollination systems and distribution ranges (endemism) of plant species from different herbaceous-shrubby communities, and how such associations may be interrelated with the incidence of reproductive strategies promoting outbreeding or inbreeding at the community level.

## Study Areas

**Fig. 1.**
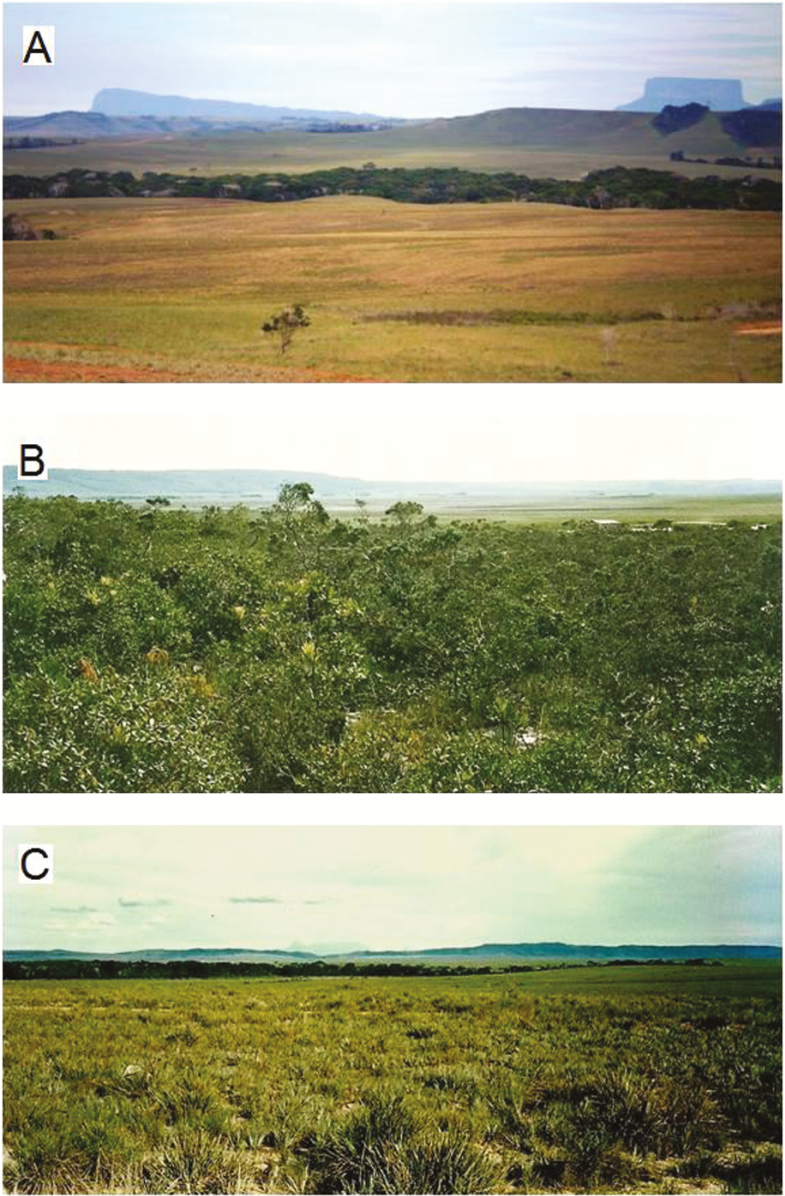
Photograph of the northern part of the Gran Sabana landscape (A), mesothermic shrubland (B) and savanna area studied (C).

The Gran Sabana is located on an elevated plateau (800–1500 m) in the Canaima National Park, in southeastern Venezuela (Fig. 1A). The Gran Sabana belongs to the Central Guayana Province of the Guayana Region (Huber 1994) and the expected vegetation type is an evergreen mountain forest according to the climate regime (Huber 1995). The climate of this region has been considered as seasonally humid, with a 3-month dry season (January–March) with 100-mm rainfall (Ramírez *et al.* 1988). However, this period cannot be considered as a pronounced dry season as some rainfall, a minimum of 60 mm, occurs from January to February with maximum rain occurring in August. In addition, annual precipitation rates vary between 1815 and 3400 mm year^−1^, and the mean monthly temperature does not vary drastically throughout the year: 19.9–21.4 °C (Ramírez *et al.* 1988). The soils are in an advanced state of weathering and are characterized by low pH, low basic cations, accumulation of acidic cations and a low Ca/Al ratio in the soil solution (Dezzeo and Fölster 1994; Fölster *et al.* 2001; Ramírez *et al.* 2007).

**Fig. 2.**
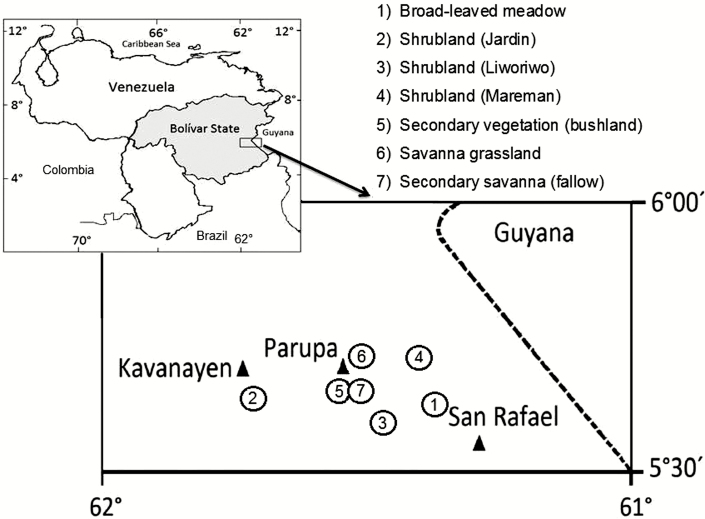
Geographical locations studied. Black triangles indicate indigenous populations. Numbers in the circles represent plant communities studied.

The fieldwork was carried out at 1241–1387 m in the northern part of the Gran Sabana Plateau (Fig. 1A) in five different herbaceous-shrubby community types (5°36′–5°44′N and 61°22′–61°41′W) (Fig. 2): shrublands (three different localities), broad-leaved meadow, savanna, secondary savanna (a fallow) and secondary vegetation [bushland (disturbed land but still retains a predominance of the original floristic and structure (Draper and Richards 2009))]. These vegetation types were the most representative herbaceous-shrubby communities on the plateau (Ramírez *et al.* 2012). The shrublands have a physiognomy and floristic composition typical of white-sand associations in Guayana (Fig. 1B), dominated by herbs and shrubs and a few small trees (Ramírez *et al.* 1988) growing on sandy soils that are nutrient poor and have low water-retaining properties (Dezzeo and Fölster 1994). They are surrounded by *Trachypogon* savanna (Fig. 1C) and frequently associated with broad-leaved meadows (Ramírez *et al.* 1988). The savanna is a typical grassland community (Fig. 1C), distributed on flat or sloping areas with sandy loam, acidic and nutrient-poor soils, dominated by perennial and annual herbs with *Trachypogon spicatus* being the most abundant species (Ramírez *et al.* 2007). Broad-leaved meadows occur on a peat substrate, which originated by soil compaction and low water permeability of hydromorphic horizons (Dezzeo and Fölster 1994). They are dominated by herbaceous species, with the main distinctive plant families being Xyridaceae, Rapateaceae and Eriocaulaceae. *Stegolepis ptaritapuiense* (Rapateaceae) is the most frequent species. Secondary bushland was represented by the re-growth of a forest deeply disturbed by anthropogenic activity and dominated by perennial herbs, shrubs and annual herbs, followed by trees and climbers (Ramírez *et al.* 2012) growing on clayey, acidic and nutrient-poor soils (Dezzeo and Fölster 1994). A secondary savanna, a fallow represented by an abandoned area of cultivated, uncultivated and disturbed grassland, was also studied. The fallow is dominated by herbaceous species, mainly pioneer species (Ramírez *et al.* 2012). All study areas are located on an area of ~40 km^2^ and therefore climate as a factor in the differences across communities was discounted.

## Methods

### Endemism

Plant species were classified as endemic or non-endemic according to information published in the Flora of the Venezuelan Guayana (Steyermark *et al.* 1995–2005) and the New Catalog of the Vascular Flora of Venezuela (Hokche *et al.* 2008). Data about endemic plant species come from Ramírez *et al.* (2012). Most of the endemic species are restricted to the Venezuela Guayana (e.g. *Ilex subrotundifolia*, *Calea nana*, *Pagameopsos garryoides*, *Remijia densiflora* and *Smilax pittieriana*). However, some endemic species studied (e.g. *Calliandra pakaraimensis* and *Cleistes unifoliate*) are endemic to the Guiana Shield, which underlies Guyana, Suriname, French Guiana and most of Venezuela, as well as parts of Colombia and Brazil (Gibbs and Barron 1993). Some of these plant species have been described recently from neighbouring countries (Hokche *et al.* 2008).

### Plant life-forms and succulence

Plant life forms were established according to habit, longevity, stem lignification, height and type of ramification. At first, plant species were classified as perennial and short-lived species. Short-lived condition was established in herbaceous species observing a minimal of 10 individuals per species during 2 years in permanent plots. Those species where more than 80 % of the individuals died during the observation period were considered as short-lived or annual species. According to this, plant species were classified as tree, shrub, liana, perennial herb and annual herb (Ramírez *et al.* 2012). Species were also classified into two categories as succulents, having specialized fleshy tissue in a plant organ for the conservation of water, and non-succulents.

### Substrate type

Plant species were also classified in accordance with the type of substrate where plant species grew: (i) terrestrial plants, plant growing on the soil, with roots anchored directly in the soil, and (ii) epiphytes, plant anchored on other plants. The second group includes parasite, hemiparasite, which obtain water and nutrient from the host plant and non-parasite species. Data about substrate type relations come from previous studies (Ramírez *et al.* 2012).

### Nutritional relations

Plant species were classified pertaining to the nourishment of organisms into three categories: (i) autotroph, plant that makes its own food, (ii) hemiparasite and parasite, partial or total parasite that obtains its nourishment from a living plant and (iii) insectivore, plant that obtains partially its nourishment from insects. Parasitism, hemiparasitism and insectivory were mostly inferred by the modification of habit, leaves and roots, and then confirmed with systematic studies (Smith *et al.* 2004). Information about nutritional relations comes from previous studies (Ramírez *et al.* 2012).

### Carbon metabolism

Plant species were classified according to three main pathways of carbon assimilation, C_3_, C_4_ and CAM, following data previously published (Ramírez and Briceño 2015; Ramírez and Herrera 2017).

### Successional stages

Plant species were also sorted in relation to the successional status where plant species grew in each community: (i) late seral or climax species and (ii) pioneer species. In the first case, late seral species were found growing in natural or undisturbed areas, and pioneer, were found growing into areas disturbed, like edge road, and mainly disturbed areas created by fire and agricultural machinery.

### Pollination systems

Information about pollinator agents comes from unpublished data by N. Ramírez, which were determined following Ramírez (2004). Observations were made over 24 months during a 5-year period. The activity of all types of floral visitors was described and then the visitors were captured. Pollinators were distinguished from floral visitors using five qualitative criteria.

Information about plant pollination systems was established according to the criteria of specialization. Plant species were categorized according to their pollination system specificity in relation to their pollen vectors (slightly modiﬁed from Faegri and van der Pijl 1979). In this study, the following categories were used: (i) polyphily, pollinated by different taxonomic orders of visitors, (ii) oligophily, pollinated by more than one family of the same taxonomic order of visitors and (iii) monophily, pollinated by only one species, one genus or different genera of the same taxonomic family. The occurrence of wind pollination was recognized according to floral morphology (Faegri and van der Pijl 1979) and in some cases tested by enclosing flowers or inflorescences in 1-mm nylon mesh bags which excluded most insects but allowed the passage of airborne pollen (Bawa and Krisp 1980).

### Sexual systems

Plant species were categorized as hermaphroditic, andromonoecious, gynomonoecious, monoecious, subdioecious or dioecious based mainly on morphological and functional criteria. Some morphologically hermaphroditic species were considered andromonoecious due to the absence of ovules in at least 20 % of the flowers (Ramírez 2005*a*). Additionally, on the basis of breeding systems under controlled crosses, some morphologically hermaphrodite species were considered dioecious. For comparative analyses, only three categories, hermaphrodite, monoecy (including andromonoecious and gynomonoecious species) and dioecy (including androdioecious, gynodioecious and distylous-functional dioecious species), were considered.

### Herkogamy

The spatial separation or the non-spatial separation of pollen presentation and pollen receipt within flowers of hermaphrodite species and hermaphrodite functional-dioecious species or between flowers of monoecious were determined. Herkogamy was determined when the stigma was positioned at a statistically significant separation from the anther. In this study, the occurrence of ordered herkogamy was examined (Webb and Lloyd 1986). Plant species were classified as herkogamous and non-herkogamous.

### Temporal variation in sexual expression

Temporal variation in sexual expression was determined following Ramírez (2005*a*). All hermaphroditic, submonoecious, monoecious and hermaphrodite functional-dioecious species were examined to determine whether individual flowers or inflorescences (when treated as pollination units) had synchronous or asynchronous male and female phases (Lloyd and Webb 1986). In most of the species, sexual expressions synchrony was determined by observations at 2-h intervals from the start of anthesis until flower or inflorescence marcescence, in a minimum of 10 flowers or inflorescences per species. Maturation of the stamens was determined by anther dehiscence or, in the case of poricidal anthers, by the time when pollen could be dislodged from anthers. Female maturity was determined by a shiny or moist stigmatic surface in taxa with wet stigmas, or by the elongation of the style and full development of the stigma in taxa with dry stigmatic surfaces. Plant were categorized as adichogamous (sexual synchrony, following Faegri and van der Pijl 1979), protandrous (anther dehiscence occurring before stigmatic receptivity) or protogynous (stigmatic receptivity prior to anther dehiscence). The latter two categories may include species with posterior overlapping of the sexual phases (incomplete dichogamy, *sensu*Lloyd and Webb 1986).

### Experimental pollination tests

Reproductive efficiency under experimental conditions was determined at two levels: (i) fruits developed per total number of flowers and (ii) total number of non-abortive seeds produced by all fruits per total number of ovules (flower number multiplied by average number of ovules per flower). The experimental pollination tests considered in this study were: (i) agamospermy test, as fruits and/or seeds produced from emasculated and isolated flowers; (ii) spontaneous self-pollination test, as fruits and/or seeds produced from isolated and non-manipulated flowers; (iii) self-pollination test, as fruits and/or seeds produced from hand or assisted self-pollinated flowers and (iv) cross-pollination test, as fruits and/or seeds produced from hand outcrossed flowers. Breeding system data for Melastomataceae and *Dimorphandra macrostachya* were taken from previous studies (Hokche and Ramírez 2008; Ramírez and Briceño 2016).

### Breeding system indexes

Four breeding system indexes (BSI) were determined at fruit and/or seed level following Ramírez and Nassar (2016). Each BSI results from the quotient of two contrasting experimental tests, where the denominator is expected to be the largest referential value. In the case where the conclusion derived from both fruit and seed level differed, we opted for the conclusion obtained at seed level.

The Index of Agamospermy (IAG) was determined dividing the results obtained from the agamospermy test by the results obtained from the cross-pollination test (Riveros *et al.* 1996); however, because reproductive efficiency of self- and cross-pollination tests may or may not be different in agamospermous species, the IAG had to be calculated on the basis of both cross- [IAG(cp)] and self-pollination [IAG(sp)] tests. Between the two, the index with the lowest value is the most appropriate one to be used, because it represents the comparison of agamospermy against the most efficient pollination test (Ramírez and Nassar 2016). The Index of Spontaneous Self-Pollination (ISSP) or Automatic Self-pollination Index (Ruiz-Zapata and Arroyo 1978; Sobrevila and Arroyo 1982) was determined dividing reproductive efficiency from the spontaneous self-pollination test by reproductive efficiency obtained from the assisted self-pollination test. The Index of Self-Fertility (ISF) (Lloyd and Schoen 1992) was determined dividing the results from the spontaneous self-pollination test by results from the cross-pollination test. Finally, the Index of Self-Incompatibility (ISI) or Genetic Self-incompatibility Index (Ruiz-Zapata and Arroyo 1978; Sobrevila and Arroyo 1982) was determined dividing the results from the hand self-pollination test by results from the cross-pollination test.

### Breeding index categories

Five categories for each BSI were used (Ramírez and Nassar 2016): (i) BSI = 0, (ii) 0 < BSI < 1.0, (iii) BSI = 1.0, (iv) 0 < (1/BSI) < 1.0 (when BSI > 1.0) and (v) 1/BSI ~ 0 (when BSI ~ ∞). This system of categories is a symmetrical model at both sides of value 1.0, positioning contrasting categories at the extremes: 0 (BSI= 0) and ∞ (1/BSI ~ 0) values, which represent opposite biological conditions. Intermediate values, below (0 < BSI < 1.0) and above (0 < (1/BSI) < 1.0) 1.0, but lower than the extreme conditions, correspond to intermediate or transitional biological categories. BSI = 1.0 denotes the referential value, indicating that the experimental tests conforming the index render approximately equal results. More details about categorization of the BSI, assumptions and exceptional cases may be consulted in Ramírez and Nassar (2016).

In addition, some species where spontaneous self-pollination is avoided because morphological traits, sexuality and dichogamy were considered as non-spontaneous self-pollination (BSI = 0): (i) plant species having pollen grouped into masses, pollinia, which have to be move by pollinators from the androecium to the stigma (Asclepiadoideae in the Apocynaceae and Orchidoideae and Epidendroideae in the Orchidaceae) and (ii) monoecious–hercogamous–dichogamous species where unisexual flowers occur separately in time and space, without any possibility of spontaneous self-pollination (*Philodendron*, *Phyllanthus*, *Phoradendron*).

### Statistical analysis

We used a *t*-test; with degrees of freedom equal to *n* − 1 (Sokal and Rohlf 1998), to discriminate between BSI values from 0 and 1.0 (see Ramírez and Nassar 2016 for details). When BSI values were higher than ≥1.0 (up to infinite), the inverse value (1/BSI) was used instead of BSI, to make the statistical method symmetrical at both sides of BSI = 1.

To establish the level of dependence between reproductive variables and functional groups, log-linear analyses of frequency were performed using two-way tables (Statsoft 2007). The concept of interaction in log-linear analyses is analogous to that used in analysis of variance. When the log-linear analysis of frequency was significant, residual frequencies, that is, observed minus expected frequencies, were estimated for each cell of the two-factor comparison, and then standardized and tested for significance. This analysis allowed us to establish which pairs of variables deviated significantly from expected values (Legendre and Legendre 1993), and therefore, made a larger contribution to the association. Significant and positive residuals indicated a strong association between both categories, and significant and negative residuals indicated an unusual occurrence.

## Results

### Sexual system, dichogamy and herkogamy

Information on plant species, taxonomic position, sexual systems, dichogamy, herkogamy, successional stage and plant communities are compiled in **Supporting Information—Appendix 1**. Of the 348 plant species studied in the seven herbaceous-shrubby communities, 257 (73.8 %) were hermaphroditic, 59 (16.9 %) were monoecious and 32 (9.2 %) were dioecious. Hermaphrodite species were mostly homomorphic (*N* = 247, 70.97 %); few were distylous species (*N* = 10, 2.9 %). Monoecious species (*N* = 59, 17.0 %) included 6.9 % (*N* = 24) andromonoecious, 4.3 % (*N* = 15) gynomonoecious and 5.7 % (*N* = 20) had unisexual flowers only. Andromonoecy was well represented in Poaceae and Cyperaceae, and gynomonoecy in Asteraceae **[see****Supporting Information—Appendix 1****]**. Most dioecious species (*N* = 32, 9.2 %) were morphologically hermaphrodites, functional dioecious (8.1 %; *N* = 28), and only four species (1.1 %) had morphologically unisexual flowers. The largest part of the subdioecious species was hermaphrodite heteromorphic species (*N* = 13, 3.7 %), which can have rudimentary or vestigial sexual organs, followed by androdioecy (*N* = 12, 3.4 %). The lowest frequency of sexual forms was hermaphrodite distylous, gynodioecious and androgynodioecious species accounting each one (*N* = 1, 0.3 %).

### Herkogamy and dichogamy

Frequency of adichogamous (*N* = 194; 61.4 %) was higher than dichogamous (*N* = 122; 38.6 %) hermaphrodite and monoecious species examined. Protandry (*N* = 86; 27.2 %) was higher than protogyny (*N* = 36; 11.4 %). Herkogamous, including subdioecious species (*N* = 265; 76.8 %), was higher than non-herkogamous species (*N* = 80; 23.2 %). The relationship between temporal variation in sexual expression and herkogamy was statistically significant (χ^2^ = 22.4, *P* < 0.00001). Herkogamous species were mostly adicogamous (*N* = 163; 68.2 %), followed by protandrous (*N* = 49; 20.5 %) and protogynous (*N* = 27; 11.3 %) species, respectively.

The relationship between sexual system and herkogamy was statistically significant (χ^2^ = 9.56; *P* < 0.008). Most of the hermaphrodite were herkogamous species (*N* = 202; 78.6 %) and a lesser frequency of monoecious species was herkogamous (*N* = 37; 62.7 %). The main part of the morphologically hermaphrodites flowers of subdioecious species were herkogamous (*N* = 26; 89.7 %). Temporal variation in sexual expression and sexual system (hermaphrodite and monoecy only) was also significantly associated (χ^2^ = 82.6; *P* < 0.0000001). Hermaphrodite species accounted 72.4 % (*N* = 186) of adichogamy, 22.2 % (*N* = 57) of protandry and 5.4 % (*N* = 14) of protogyny. In contrast, most of monoecious were dichogamous species (*N* = 51; 87.9): 50 % (*N* = 29) were protandrous, 37.9 % (*N* = 22) were protogynous and 12.1 % (*N* = 7) were adichogamous species.

### Sexual systems and associated attributes

Plant sexual system was significantly associated with life form, succulence, substrate type, nutritional relations, carbon metabolism, successional stage, pollination systems and endemism (Table 1). The proportion of hermaphrodite species was similarly distributed for shrubs, liana and perennial herbs, lower for trees and higher for annual species. Monoecious species were most frequent in perennial herbs, and lower for shrubs and liana. The proportion of dioecious species was higher for trees and lowers for annual herbs, and was significantly associated with shrubs. The proportion of dioecious species was higher for succulent than non-succulent species; the contrary was found for monoecy. Monoecious and dioecious species frequencies were higher in epiphyte than in terrestrial species. The proportions of monoecy and dioecy were higher for parasitic–hemiparasite than for autotroph and insectivore species. Dioecious species were more frequent in late than pioneer stages. The lowest proportion of hermaphrodite species and the highest proportion of monoecious species were C_4_ species. Monoeciuos species were significantly associated with anemophily, and most of dioecious species were polyphilous species. Hermaphroditism and monoecism were higher for non-endemic species. In contrast, dioecy was the most frequent sexual system for endemic species **[see****Supporting Information—Table S1****]**.

**Table 1.** Results of chi square tests (χ^2^) and residual analyses for the association between reproductive and non-reproductive traits. Characters link by a hyphen indicates association statistically significant for the residual analysis. Superscript indicates: ^a^positive residual at *P* < 0.05; ^b^positive residual at *P* < 0.01; ^d^negative residual at *P* < 0.01. Positive residuals indicated a strong association and negative residuals indicated an unusual occurrence. Life form: S, shrub. Carbon metabolism: C_4_. Pollination system: O, oligophily; A, anemophily. Endemism: E, endemic. Sexual system: D, dioecy; M, monoecy. Dichogamy: Ad, adichogamy; Prot, protandry; pg, protogyny. Herkogamy: NH, no herkogamy.

	Reproductive trait					
	Sexual system		Dichogamy		Herkogamy	
	χ^2^(*P*<)	Residual analysis	χ^2^(*P*<)	Residual analysis	χ^2^(*P*<)	Residual analysis
Life form	68.6 (0.001)	D-S^a^	21.8 (0.005)	-	31.1 (0.001)	-
Succulence	31.1 (0.001)	-	1.3 (n.s.)	-	0.47 (n.s.)	-
Substrate type	11.2 (0.004)	-	1.6 (n.s.)	-	2.1 (n.s.)	-
Nutritional relations	17.7 (0.001)	-	11.6 (0.020)	-	2.4 (n.s.)	-
Carbon metabolism	24.2 (0.001)	M-C_4_^b^	46.1 (0.001)	Ad-C_4_^d^; Prot-C_4_^b^	28.6 (0.001)	NH-C_4_^b^
Successional stage	9.2 (0.010)	-	2.9 (n.s.)	-	0.1 (n.s.)	-
Pollination system	56.5 (0.001)	M-O^d^; M-A^b^	76.5 (0.001)	Ad-A^d^; Prot-A^b^; Pg-A^a^	40.0 (0.001)	NH-A^b^
Endemism	9.9 (0.007)	D-E^a^	1.6 (n.s.)	-	2.1 (n.s.)	-
Community	20.46 (n.s.)	-	22.4 (0.034)	-	25.7 (0.001)	-

### Dichogamy and associated attributes

Temporal variation in sexual expression was significantly associated with life form, nutritional relations, carbon metabolism and pollination systems (Table 1). The proportion of protandry was higher for perennial herbs, annual herbs and trees, and protogyny was most common in perennial herbs. The lowest values of adichogamous species were found in perennial herbs. Most of the insectivore species were adichogamous, and the highest frequency of protandry corresponded to parasitic–hemiparasitic species. The highest proportions of dichogamous and the lowest of adichogamous were found in C_4_ species, respectively. In the first case, positive residual indicated a strong association between protandrous and C_4_ species; in the second case, negative residuals indicated an unusual association between adichogamous and C_4_ species (Table 1). Similar trends were found between anemophily and temporal variation in sex expression: most protandrous and protogynous species were anemophilous. Positive residuals revealed a strong association between protandry and anemophily, and negative residuals indicated an unusual association between adichogamy and anemophily. In addition, the proportion of protandrous species was high for monophily and comparatively lower for anemophily **[see****Supporting Information–Table S1****]**.

### Herkogamy and associated attributes

Herkogamy was significantly associated with life form, carbon metabolism and pollination systems (Table 1). The proportion of herkogamy was higher for trees, shrubs and liana, and the lowest values were found in herbaceous species. Most of the C_4_ species were non-herkogamous. The proportion of herkogamous species was higher for monophilous and lesser for anemophilous species, where positive residual indicated a strong association between anemophily and non-herkogamy **[see****Supporting Information—Table S1****]**.

### Sexual traits and plant communities

**Fig. 3.**
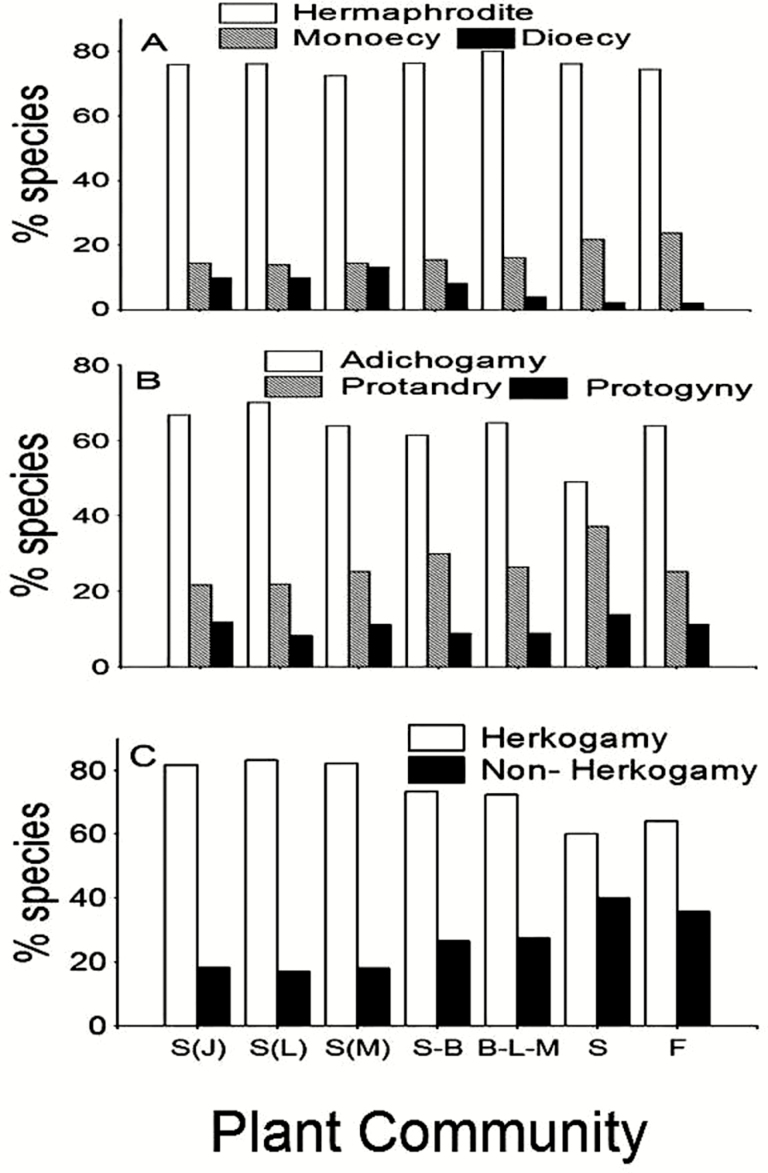
Frequency distribution of sexual system (A), temporal variation in sexual expression (B) and spatial separation of sexual organ (C) according to plant community: S(J), shrubland (Jardin); S(L), shrubland (Liworiwo); S(M), shrubland (Mareman); S-B, secondary bushland; B-L-M, broad-leaved meadow; S, savanna; F, fallow.

Temporal variation in sexual expression and spatial sexual separation was significantly associated with plant community, and sexual system was not significantly associated with plant community; however, some interesting trends were observed **[see****Supporting Information—Table S1****]**. The proportion of hermaphrodite species was similarly distributed across plant communities. Monoecy was slightly higher for savanna and fallow than the other communities, and dioecy was higher for shrublands and secondary bushland than savanna and fallow (Fig. 3A). Temporal variation in sexual expression shows that the lowest and highest frequency of adichogamy and dichogamous species, respectively, were found in the savanna. The highest proportion of protogyny was also found in shrublands, secondary bushland and fallow (Fig. 3B). The highest proportion of herkogamous species occurred in the shrublands and the lowest in the savanna and fallow (Fig. 3C).

### Breeding systems

Information about flowers number, fruit and seed produced under experimental tests for 103 plant species belonging to 40 plant families of herbaceous-shrubby communities from the Gran Sabana Plateau is detailed in **Supporting Information—Appendix 2**. Breeding system indexes and their qualitative categories are detailed in **Supporting Information—Appendix 3**.

### Breeding systems and associated attributes

#### Agamospermy

Most plant species studied were non-agamospermous (IAG = 0; *N* = 75), followed by partially agamospermous species (0 < IAG < 1.0; *N* = 16), and partially constrained sexual mating (0 < (1/ IAG) < 1.0; *N* = 2). There were not found fully agamospermous species. The percentage of plant species according to functional groups ranged from 75 to 100 % for non-agamospermous species, except for insectivore with only one partially agamospermous species and parasitic–hemiparasitic with 50 % of non-agamospermous and 50 % of partially agamospermous species **[see****Supporting Information—Table S2****]**. More than 20 % of trees, shrubs and annual herbs were partially agamospermous and less than 2 % of shrubs and perennial herbs had partially constrained sexual mating. The succulent group shows that only 18.6 % of non-succulent species were partially agamospermous, and one succulent and one non-succulent species had partially constrained sexual mating. All epiphytes were non-agamospermous species and 17.8 % of terrestrial plants were partially agamospermous; two terrestrial species had partially constrained sexual mating. All C_4_ and CAM species were non-agamospermous. The relationship between agamospermy and pollination system showed that all anemophilous were non-agamospermous. Values close to 20 % of monophilous and oligophilous species were partially agamospermous, and <5 % of monophilous and polyphilous species were partially constrained sexual mating. All endemic species were non-agamospermous.

**Fig. 4.**
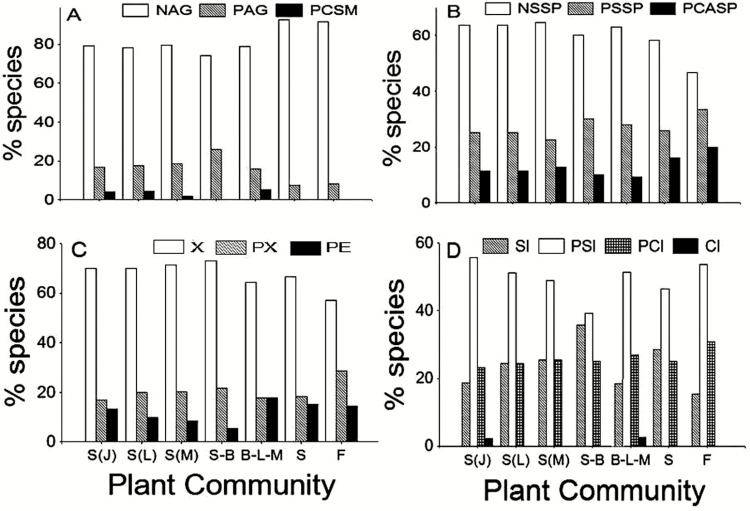
Frequency distribution of breeding system categories according to plant community: S(J), shrubland (Jardin); S(L), shrubland (Liworiwo); S(M), shrubland (Mareman); S-B, secondary bushland; B-L-M, broad-leaved meadow; S, savanna; F, fallow. Agamospermy index categories: NAG, non-agamospermous; PAG, partially agamospermous; PCSM, partially constrained sexual mating (A). Spontaneous self-pollination index categories: NSSP, non-spontaneous self-pollinated; PSSP, partially spontaneous self-pollinated; PCASP, partially constrained assisted self-pollination (B). Self-fertility index categories: X, xenogamous; PX, partially xenogamous; PE, partially endogamous (C). Self-incompatibility index categories: SI, self-incompatible; PSI, partially self-incompatible; PCI, partially cross-incompatible; CI, cross-incompatible (D).

The majority of species examined in the seven communities were non-agamospermous, from 74.2 to 92.6 %. The highest frequency of non-agamospermous species was found in areas of savanna, relatively natural and deeply disturbed like fallow, and the lowest was found in the secondary bushland; intermediate values were recorded in the three shrublands and broad-leaved meadow (Fig. 4A). Partially agamospermous species frequency ranged from 8.3 to 25.8 % among plant communities: the highest frequency was found in the secondary bushland, the lowest in the savanna and fallow and intermediate values were found in the shrublands. Partially constrained sexual mating was the less frequent category of the agamospermy index, and varied from 0.0 to 5.3 %. The highest values were registered in the broad-leaved meadow and in two of the three shrublands **[see****Supporting Information—Table S2****]**.

#### Spontaneous self-pollination

Three categories of the ISSP were recorded: non-spontaneous self-pollinated (ISSP = 0; *N* = 72), partially spontaneous self-pollinated (0 < ISSP < 1.0; *N* = 28) and the lowest values was found for partially constrained assisted self-pollinated species (0 < (1/ISSP) < 1.0; *N* = 11). *Mandevilla benthamii*, *Polygala longicaulis* and *Echinolaena inflexa*, statistically considered partially spontaneous self-pollinated, have values of ISSP close to non-spontaneous self-pollinated category **[see****Supporting Information—Appendix 2****]**.

The percentage of plant species according to functional groups, ranged from 60 to 100 % for non-spontaneous self-pollination species, except for insectivores, with only one assisted self-pollination partially constrained species, annual herbs with one non-spontaneous self-pollination species, and anemophilous, with three non-spontaneous self-pollination species **[see****Supporting Information—Table S2****]**. All lianas were non-spontaneous self-pollination species, 22–29 % of tree, shrub and perennial herb and 50 % of annual species were partially spontaneous self-pollination. Partially constrained assisted self-pollination species were more frequent among annual herbs, followed by shrubs and perennial herbs. Succulent species had one partially spontaneous self-pollination and one assisted self-pollination partially constrained species. Terrestrial were more numerous than epiphytic species for partially self-pollination and partially constrained assisted self-pollination, respectively; however, partially self-pollination was similar for terrestrial and epiphytic. Despite non-significant relations between spontaneous self-pollination index categories and successional stages, the frequency of partially constrained assisted self-pollination was more than twice higher in pioneer than late stage species. The frequency of partially spontaneous self-pollination was higher for C_3_, intermediate for C_4_ and lowest for CAM species. Spontaneous self-pollination categories and pollination system showed that partially spontaneous self-pollination species ranged from 22 to 25 % for biotic pollination system and 55.6 % for wind-pollinated species.

Non-spontaneous self-pollinated species varied from 46.7 to 64.5 % among plant communities (Fig. 4B). The highest frequencies were found in the shrublands and the lowest in the savanna and fallow. Partially spontaneous self-pollination frequency was lower in the shrubland (Mareman) and higher in the fallow. Partially constrained assisted self-pollination category was higher in the savanna and fallow than in the other plant communities.

#### Self-fertility

Three categories of the ISF were recorded: xenogamous (ISF = 0; *N* = 76), partially xenogamous (0 < ISF < 1.0; *N* = 19) and partially endogamous (0 < (1/ISF) < 1.0; *N* = 11) species. *Rhynchospora pilosa* and *Chelonanthus angustifolius*, statistically considered partially endogamous, have self-fertility indexes close to autogamous species. In a similar way, *Ternstroemia crassifolia*, statistically considered partially xenogamous, has self-fertility index close to xenogamy **[see****Supporting Information—Appendix 3****]**.

Xenogamous species ranged from 60 to 100 % according to functional groups, except for insectivore, one annual herb (25 %) and anemophilous (50 %) species **[see****Supporting Information—Table S2****]**. The highest frequency of xenogamy was found in trees, lianas and shrubs. The frequency of partially xenogamous and partially endogamous species was higher for herbaceous species. Partial endogamy occurred approximately twice for pioneer compared with late stage species. Partially xenogamous species frequency was higher for wind pollination than for biotic pollination systems; the lowest frequency was found in monophilous and polyphilous species. Partially endogamous species was similarly distributed across pollination system specificity.

Xenogamous species were the most common breeding category at the community level, which varied from 57.1 % in the fallow to 70–73 % in the shrublands and secondary bushland (Fig. 4C). Partially xenogamous species were found lower in the shrublands (Jardin), broad-leaved meadow and savanna, and higher in the fallow. Partially endogamous species was lower in the secondary bushland and shrubland (Mareman) and higher in the broad-leaved meadow, savanna and fallow.

#### Self-incompatibility

Four categories of the ISI were found in the sample examined: partially self-incompatible (0 < ISI < 1.0; *N* = 36), self-incompatible (ISI = 0; *N* = 27), partially cross-incompatible (0 < (1/ISI) < 1.0; *N* = 18) and only one species was cross-incompatible (*Phyllanthus majus*). Three plant species, *Meriania sclerophylla*, *Tococa nitens* and *Nietneria paniculata*, considered partially self-incompatible, have self-incompatibility indexes closely to self-compatible species **[see****Supporting Information—Appendix 3****]**.

The relationship between the categories of ISI and functional groups are detailed in **Supporting Information—Table S2**. Trees and liana were predominantly self-incompatible, herbaceous species were mostly partially self-incompatible and from 20 to 33 % of shrub and herbaceous species were partially cross-incompatible. Most of succulent species were partially self-incompatible, followed by partially cross-incompatible. All insectivore, and parasitic–hemiparasitic, and the majority of C_4_ and CAM were partially self-incompatible species. Partially self-incompatible species frequency was higher for wind pollination than for biotic pollination systems; the lowest value was found in monophilous species; oligophilous and polyphilous species exhibited values of partially self-incompatible close to 50 %. The largest frequency of partially cross-incompatibility was found for monophilous species.

Partially self-incompatible species were the most common breeding category at the community level, which varied from 39.2 % in the secondary bushland to 53.8–55.8 % in the fallow and shrubland (Jardin) (Fig. 4D). Self-incompatible species were found lower in the fallow and broad-leaved meadow, and higher in the secondary bushland. Therefore, the frequency of self-incompatibility and partial self-incompatibility was closely related in the secondary bushland. Partially cross-incompatible species was higher in the fallow and very similar between the other communities. Cross-incompatibility was the lowest frequency of self-incompatibility index and was only found in the shrubland (Jardin) and broad-leaved meadow.

### Breeding system and sexuality, dichogamy and herkogamy

The relationship between sexual traits and breeding systems was not significant; however, some trends are detailed in Table 2. Most of the agamospermous species were found for hermaphrodite, monoecious, herkogamous, adichogamous and protandrous species; partially agamospermous were hermaphrodite, herkogamous and adichogamous species. Likewise, the largest frequency of non-spontaneous self-pollinated was hermaphrodite, monoecious, herkogamous, adichogamous and protandrous species. Partially spontaneous self-pollinated were mainly hermaphrodite, herkogamous, adichogamous and dichogamous species; partially constrained assisted self-pollinated were hermaphrodite, herkogamous, adichogamous and protandrous species. Self-fertility index categories exhibited the same relation with the sexual traits: xenogamous were commonly hermaphrodite, monoecious, herkogamous, adichogamous and protandrous species; partially xenogamous were mostly hermaphrodite, herkogamous and adichogamous species, and partially endogamous were mostly hermaphrodite, herkogamous, adichogamous and dichogamous species. Self-incompatible and partially self-incompatible were mostly hermaphrodite, herkogamous and adichogamous species. In addition, some partially self-incompatible were also non-herkogamous and protandrous species. Partially cross-incompatible were primarily hermaphrodite, herkogamous and adichogamous species.

**Table 2.** Relationship between BSI categories and morphological and temporal organization sexual traits.

	Sexuality		Herkogamy		Dichogamy		
Breeding system	Hermaphrodite	Monoecy	Herkogamous	Non-Herkogamous	Adichogamy	Protandry	Protogyny
	N (%)	N (%)	N (%)	N (%)	N (%)	N (%)	N (%)
Agamospermy index categories							
Non-Agamospermous	65 (69.9)	10 (10.7)	67 (72.1)	8 (8.6)	48 (52.2)	18 (19.6)	8 (8.7)
Partially agamospermous	16 (17.2)	0 (0.0)	14 (15.1)	2 (2.2)	11 (12.0)	2 (2.2)	3 (3.3)
Partially constrained sexual mating	2 (2.2)	0 (0.0)	2 (2.2)	0 (0.0)	0 (0.0)	2 (2.2)	0 (0.0)
Spontaneous self-pollination index categories							
Non-spontaneous self-pollinated	61 (54.9)	11 (9.9)	68 (61.3)	4 (3.6)	49 (44.6)	16 (14.6)	6 (5.4)
Partially spontaneous self-pollinated	22 (19.8)	6 (5.4)	23 (20.7)	5 (4.5)	15 (13.6)	7 (6.4)	6 (5.4)
Partially constrained assisted self- pollination	11 (9.9)	0 (0.0)	9 (8.1)	2 (1.8)	6 (5.4)	4 (3.6)	1 (0.9)
Self-fertility index categories							
Xenogamous	62 (58.5)	13 (12.3)	70 (66.0)	5 (4.7)	51 (48.6)	17 (16.2)	6 (5.7)
Partially xenogamous	16 (15.1)	4 (3.8)	18 (16.9)	2 (1.9)	13 (12.4)	5 (4.8)	2 (1.9)
Partially endogamous	10 (9.4)	1 (0.9)	7 (6.6)	4 (3.8)	5 (4.8)	4 (3.8)	2 (1.9)
Self-incompatibility index categories							
Self-incompatible	22 (28.2)	3 (3.8)	25 (32.1)	0 (0.0)	19 (24.7)	4 (5.2)	1 (1.3)
Partially self-incompatible	32 (41.0)	4 (5.1)	27 (34.6)	9 (11.5)	21 (27.3)	12 (15.6)	3 (3.9)
Partially cross-incompatible	15 (19.2)	2 (2.6)	15 (19.2)	2 (2.6)	11 (14.3)	3 (3.9)	3 (3.9)

## DiscusAsion

Outbreeding and inbreeding strategies recognized in this study are associated with functional groups and isolation of the herbaceous-shrubby communities in the Gran Sabana Plateau. Dioecy and monoecy, together dichogamy and herkogamy, varied according to life forms, pollination systems specificity, successional stage, endemism and vegetation structure. High levels of outbreeding strategies, non-agamospermy, non-spontaneous self-pollination, xenogamy and partial self-incompatibility, occurred mainly in woody species from shrublands and secondary bushland. Inbreeding strategies, non-herkogamy, spontaneous self-pollination and partial cross-incompatibility, are primarily associated with herbaceous life forms from disturbed communities. In this context, ecological and evolutionary trends of mating systems of the herbaceous-shrubby communities in the Gran Sabana Plateau are discussed.

### Sexual systems

The proportion of hermaphrodite species was similarly distributed across herbaceous-shrubby communities in the Gran Sabana Plateau. In contrast, there is a clear trend in the frequency of dioecy, life form and plant community; high for woody species in more structured communities, shrublands and secondary bushland and lesser for herbs in herbaceous communities, savanna and fallow; the opposite occurred for monoecious species. The relationship between life form and plant community in the Gran Sabana Plateau (Ramírez *et al.* 2012) and the association between sexual system and life form suggest that the frequency of life forms observed in the sample could be considered the proximate cause in the distribution of dioecy and monoecy in the shrubby-herbaceous communities. The highest frequency of dioecy in shrubby communities is related to the association between dioecy and woody condition (Vary *et al.* 2011; Schlessman *et al.* 2014; Godin 2017). However, the frequency of dioecy in the shrubby communities studied was lower than in tropical forests because the low frequency of woody species in the communities studied. In addition, separate sexes are favoured in stressful environments (Matallana *et al.* 2005). The proportion of dioecy in shrubby communities and monoecy in herbaceous communities may be also associated with the poor soil condition in the Gran Sabana Plateau, where the soils are in an advanced state of weathering and are characterized by low pH and deﬁciency of basic cations (Dezzeo and Fölster 1994; Fölster *et al.* 2001; Ramírez *et al.* 2007). Therefore, poor soil condition may be also associated with the occurrence and evolution of dioecy and monoecy in these communities.

Dioecy and monoecy were found in non-woody epiphyte and parasite-hemiparasite species, where the frequencies were larger than 30 %. Dioecy and monoecy in epiphyte–parasite–hemiparasite species may be related with the occurrence of dicliny in the main families of parasite–hemiparasite species in the study area, Santalaceae (Miller 2005) (including Loranthaceae (Kuijt 2001)) and Viscaceae (Kuijt 2005), which are phylogenetically related (APG III 2003). Therefore, dicliny in these epiphyte–parasite–hemiparasite families has a phylogenetic component. However, ecological characteristics of dioecy and monoecy in epiphyte–parasite–hemiparasite species could also be associated with the foliage being located at the same height of the trees and shrubs, and therefore under selective pollination services similar to those acting on the woody species. Parasite–hemiparasite species may also be under similar physiological conditions of host woody plants, which could enhance the same opportunity for a unisexual system than woody species. Dioecious succulent species is also influenced phylogenetically because the largest part belongs to a plant family, Clusiaceae, predominantly dioecious (Pipoly *et al.* 1998), which is a large plant family in the study areas (Pipoly *et al.* 1998; Ramírez *et al.* 2012). However, most of the plant families containing dioecious species in shrubby communities, Clusiaceae (*N* = 10), Primulaceae (*N* = 4) and Aquifoliaceae (*N* = 4), belong to three different clades (APG III 2003), and therefore the total phylogenetic effect on the incidence of dioecy at the community level seem to be limited.

It has been suggested that dioecy evolved from monoecy (Renner 2014). In the herbaceous-shrubby communities of the Gran Sabana Plateau, the evolutionary route from monoecy to dioecy does not seem to occur. Our results allow hypothesize about an alternative route for monoecy and dioecy origin. Twenty-nine dioecious species identified in this study were morphologically hermaphrodite, and therefore seem to have an ancestral origin in the hermaphrodite condition. Furthermore, pollination systems seem to be also related with the evolution of dioecy from hermaphroditism. In the seven shrubby-herbaceous communities in the Gran Sabana Plateau, dioecious had the highest frequency of polyphilous species. Polyphilous pollination seems to be associated with the evolution of dioecy from an ancestral hermaphrodite sexual system (Beach 1981; Ramírez 2005*a*). The high frequency of subdioecious species in the study area suggests that the evolution of dioecy under generalist pollination systems also take place in the Gran Sabana Plateau.

Furthermore, the evolution of dioecy may be as well associated with the history of the geographic area. Dioecy is significantly associated with endemism, such as in Madagascar (Vary *et al.* 2011) and New Caledonia (Schlessman *et al.* 2014). Endemism in the Gran Sabana Plateau has been associated with a heterogeneous environment (Steyermark 1979; Duno de Stefano *et al.* 2009). Most of the endemic species are community specialized, growing on isolated communities in the Gran Sabana Plateau (Ramírez *et al.* 2012), and dioecious endemic species seem to have arisen in islands (Bernardello *et al.* 2001; Schlessman *et al.* 2014). On the basis of this evidences, the hypothesis to be tested in the future is that dioecy in endemic species may have evolved under selective forces associated with the process of speciation in the mosaic of isolated non-disturbed communities in the Gran Sabana Plateau. Moreover, separate sexes are favoured in stressful environments (Matallana *et al.* 2005), and some endemic species seem to have evolved on oligotrophic soils in the Gran Sabana Plateau (Ramírez 2005*a*). Therefore, it is suggested that evolution of dioecy in endemic species seem to occur in habitat specialized in the Gran Sabana Plateau.

Monoecy represents the sexual system promoting cross-pollination in herbaceous communities, which is primarily related with life form: monoecious species are mostly herbaceous, such as in the Venezuelan Llanos (Ramírez 2005*a*). Renner and Ricklefs (1995) suggested that across all angiosperm families, monoecy is related to abiotic pollination. The significant association between monoecy and C_4_ species suggests that monoecy, mainly submonoecy, is a strategy associated with the herbaceous life form, wind pollination and C_4_ photosynthetic pathway. Moreover, the largest plant families in the savanna and fallow are Poaceae, Cyperaceae and Asteraceae (Ramírez *et al.* 2012), belong to two different major clades: Commelinids and Asterids (APG III 2003) and contribute with the abundance of monoecy and submonoecy in these herbaceous communities. Poaceae and Cyperaceae tend to have similar habit and reproductive biology, wind pollination, including andromonoecy. In contrast, Asteraceae species are gynomonoeciuos and insect pollination. Consequently, the abundance of monoecy in the savanna and fallow is, to some extent, influenced phyllogenetically.

Frequency of monoecy in the herbaceous-shrubby communities (16.5 %) was closely related to that found in the Venezuelan Llanos (17.1 %; Ramírez 2005*a*), which could be associated with the vegetation heterogeneity in both geographic areas. The high proportion of submonoecy found among monoecious species examined (52.8 %, 19/36) agree with the results found in the Venezuelan Central Llanos (Ramírez 2005*a*) and suggests that, in many cases, monoecy might have evolved from hermaphroditism in herbaceous species. Selective forces leading towards the evolution of monoecy could be enhanced by pollination, environmental factors (Ramírez 2005*a*) and phylogenetically (Vary *et al.* 2011). The evolution of monoecy through andromonoecy is well represented in Poaceae and Cyperaceae. Male flowers in andromonoecous species may enhance male fitness by increasing pollen amount and pollen dispersal in the population and subsequently pollination efficiency, which may be related to wind pollination in most of these species. In wind-pollinated species, the pollen transport effectiveness is subject to certain levels of imprecision or difficulty for pollination success (Levin 2012). Therefore, wind pollination requires a large amount of pollen to compensate for the hazards of the pollination process, which may be fulfilled by the male flowers. Gynomonoecy may also be associated with pollination by wind (Godin 2017); however, it is mainly associated with polyphilous pollination system in the Gran Sabana Plateau. In this context, the evolution of monoecy from hermaphroditism through gynomonoecy appears to occur under polyphilous pollination system in the sample examined, mainly Asteraceae. Such pathway from hermaphroditism to monoecy through gynomonoecy has been recognized in Asteraceae (Torices *et al.* 2011). The evolution of monoecy from hermaphroditism through gynomonoecy under polyphilous pollination seems to occur to avoid inbreeding such as the evolution of dioecy from hermaphroditism under generalist pollination system (Beach 1981; Ramírez 2005*a*).

### Herkogamy and dichogamy

The frequency of herkogamy was approximately twice that of dichogamy (2.04) in the Gran Sabana Plateau; a value slightly similar (2.43) was found in the Venezuelan Llanos (Ramírez 2005*a*). The parallelism in the frequency of dichogamy and herkogamy between contrasting geographic areas suggests general patterns in the mechanisms to avoid the interference pollen–stigma and promote cross-pollination. However, the frequency of dichogamy and herkogamy varied according to the plant community in the Gran Sabana Plateau, which is associated with the relation between dichogamy and herkogamy and functional groups taking place in each plant communities.

### Herkogamy

In an ecological context, the structure of vegetation appears to be associated with the frequency of herkogamy because the relation between the highest proportion of herkogamy for trees, shrubs and liana and the lowest values found in herbaceous species. Herkogamy is an important strategy for outcrossing in the shrublands, but has a lightly lesser importance in the savanna and fallow. The abundance of non-herkogamous species in herbaceous and disturbed areas suggests that selfing strategies may represent an important adaptation for colonizing species. Most of these herbaceous-colonizing non-herkogamous species were C_4_, which may be associated with certain taxonomic groups, Poaceae and Cyperaceae, and high reproductive efficiency (Ramírez and Herrera 2017). In addition, non-herkogamy may be selected when pollination agents are unpredictable because natural reproductive efficiency may increase. The highest frequency of herkogamy for monophily and lesser frequency of herkogamy for anemophilous species suggest that non-herkogamy is high for plant species where the pollination systems, including wind pollination, become more generalist. Non-herkogamy and consequently self-pollination may facilitate reproduction under unpredictable or imprecise pollination systems. In fact, wind-pollination and non-herkogamy were strongly associated, which corresponds to plant species from natural and disturbed savanna where anemophily is the most important pollination class (Ramírez and Briceño 2014) with high reproductive efficiency (Ramírez 2005*b*).

### Temporal variation in sexual expression

Several studies point out that protandry is more common than protogyny (Bernardello *et al.* 2001; Ramírez 2005*a*). The proportion of protandry was approximately two times the proportion of protogyny in an extensive survey of intrafloral dichogamy (Bertin 1993). The frequency of protandry was 2.4 times the frequency of protogyny in the herbaceous-shrubby communities in the Gran Sabana Plateau. However, the frequency of protandry and protogyny depends on vegetation structure in the Gran Sabana Plateau: the highest frequency of protandry was noteworthy in herbaceous and secondary vegetation (savanna and secondary bushland) and protogyny may be high in contrasting vegetation types (shrublands, savanna and fallow). In this context, protandry and protogyny may act as non-rigid mechanisms to avoid self-pollination and pollen-stigma interference in less structured habitats.

The relation between functional groups and the occurrence of dichogamy may explain the incidence of dichogamy in plant communities. The highest frequency of protandry in the savanna is associated with the relation between perennial and annual herbs and protandry and protogyny, which agree with the highest proportions of dichogamous and the lowest in adichogamous found in C_4_ species, respectively. In fact, C_4_ species are plant species very common in the savanna area (Ramírez and Briceño 2015). High frequency of protandry in the secondary bushland may be partially explained by the relation between protandry and tree species, and protandry and parasitic–hemiparasitic species, which grow mostly in shrublands and secondary vegetation (Ramírez *et al.* 2012).

Barrett (2003) pointed out that dichogamy is an exceptional widespread floral strategy occurring in many outcrossing species, regardless of the pollination system. However, the higher proportion of herkogamous species was found for monophilous species and lower for anemophilous species, and the higher proportion of dichogamy for species where pollination systems become more generalist, include wind pollination. Under unpredictable and generalist pollination systems, dichogamy may reduce the possibility of self-pollination or pollen-stigma interference. The strong association between dichogamy and anemophily, mainly for herbaceous from savanna, and high frequency of herkogamy in woody species from shrublands suggest that herkogamy and dichogamy tend to be associated with different plant communities, mainly related with the abundance of life forms in each community.

### Sexual system, dichogamy and herkogamy

Sexual system, herkogamy and dichogamy, may be in such a combination that each other’s partial effectiveness is reinforced, cross-pollination promoted and pollen–stigma interferences avoided. For instance, the presence of dichogamy associated with herkogamy may be the first step in the adaptive evolution of delayed selfing to provide reproductive assurance (Freitas and Sazima 2009). The combination of monoecy and dichogamy prevented autogamy in 91.7 % of the self-compatible monoecious species in a tropical cloud forest (Ramírez and Seres 1994). Thus, monoecious species promote cross-pollination and avoid pollen–stigma interference primarily through dichogamy (87.9 %), and monoecious-adichogamous species are mostly herkogamous (62.7 %). In contrast, hermaphrodite species tend to promote cross-pollination through herkogamy (90.0 %) and less frequently through dichogamy (27.1 %). Therefore, there is only a small proportion of plant species without adaptation for cross-pollination, represented by hermaphrodite–non-herkogamous species.

### Agamospermy

The majority of species examined in the seven communities were non-agamospermous. This pattern is consistent with the observed limited occurrence of agamospermy at the community level in many ecosystems (Raduski *et al.* 2012), with available records at the family level (Firetti 2018), and others (Ramírez and Nassar 2016). The most numerically important category including asexual seed formation was partial agamospermy, which is equivalent to facultative agamospermy, where sexual and asexual seed set can occur (Richards 1997, 2003). This category may be considered a suitable and evolutionary stable reproductive strategy, because it combines sexual and asexual reproduction (e.g. Richards 1997, 2003; Firetti 2018). The highest frequency of partially agamospermous species was found in the secondary bushland and middle values in the shrublands. Such distribution is primarily associated with life form and pollination system. Partially agamospermous species in the Gran Sabana Plateau are trees, shrubs and herbs, and most of the agamospermous species have specialized pollination; monophilous and oligophilous species. Additionally, the isolation of the herbaceous-shrubby communities in the Gran Sabana Plateau may limit the process of reproduction by the separation between populations in similar communities. Accordingly, community isolation and pollination specialization may be considered as central factors modelling the incidence of agamospermy. In the species set analysed, partially constrained sexual mating had the lowest frequency, as it is also the case for Angiosperms in general (Richards 1997, 2003). The lowest frequency of partially constrained sexual mating may be initially associated with the absence of sexual stimulus for seed formation, which has been considered less advantageous genetically (Ramírez and Nassar 2016).

### Spontaneous self-pollination

Non-spontaneous self-pollinated species were the most frequent in the study areas followed by partial spontaneous self-pollination species, which are well represented among flowering plants (Vuille 1987; Richards 1997). Non-spontaneous self-pollinated species varied from the highest frequencies in the shrublands, mainly woody species, to the lowest in the savanna and fallow, mainly herbaceous species. Partially spontaneous self-pollinated and partially constrained assisted self-pollinated species in the savanna and fallow are correlated with herbaceous life forms; most of these are recognized colonizing species (Raffl *et al.* 2007; Levin 2012). In this sense, the frequency of partially constrained assisted self-pollination was more than twice as high for pioneer than late stage species. Therefore, herbaceous life form and colonizing ability of plant species are associated with different levels of spontaneous self-pollination. Additionally, plant species adapted for wind pollination dominate the first successional stages (Opler *et al.* 1980), and spontaneous self-pollination occurrence may be influenced under generalist pollination (Anderson *et al.* 1996; Lemus-Jiménez and Ramírez 2005) and wind pollination. Our results agree with these trends, which suggest that generalist pollination system may promote spontaneous self-pollination in these herbaceous communities. Pollinator limitation and autogamy are regularly common in colonizing species and establishment in isolated areas (Wheelwright *et al.* 2006). Hence, spontaneous self-pollination may be considered the main reproductive mode in colonizing species.

### Self-fertility

Xenogamy was the most common breeding strategy in the herbaceous-shrubby communities: the highest frequency was found in the shrublands and secondary bushland and the lowest in the fallow. The highest frequency of xenogamy in the shrublands and secondary bushland may be primarily associated with the highest incidence of xenogamy in woody species (Newstrom and Robertson 2005). In contrast, partially xenogamous species were more frequent in the fallow, which may be associated with the relation between partial xenogamy and herbaceous species. In addition, the highest frequency of partially xenogamous species in the fallow is also correlated with the highest frequency of anemophilous species in this community. In fact, anemophilous species have been found more important than insect pollination for invader species (Opler *et al.* 1980; Herrera *et al.* 2009), and wind pollination has been considered an important pollination mode in disturbed areas.

Partial endogamy of individuals in plant populations had not been considered previously breeding system categories (Ramírez and Nassar 2016). Partial endogamy may occur under a variety of conditions, being more frequent for taxa growing in stressful environments, with reduced pollinator service (McMullen 1987; Lemus-Jiménez and Ramírez 2005; Levin 2012), and with some specific traits, such as invasive-exotic or colonizing species (Herrera and Nassar 2009). Partially endogamous species were more frequent in the broad-leaved meadow, savanna and fallow, which is mostly related with the dominance of herbaceous species in these communities

### Self-incompatibility

The high proportion of partially self-incompatible species recorded in our study has been interpreted as evidence of the high level of reproductive success associated with mixed-mating under the current scenario of pollination service in natural ecosystems (Ramírez and Nassar 2016), and it is considered an optimal and evolutionary stable mating strategy (Harder *et al.* 2008). Outbreeding systems such as self-incompatibility have been found in endemic species (Arroyo and Squeo 1990; Bernardello *et al.* 2001). Similarly, most of endemic species were found self-incompatible or partially self-incompatible, and mainly xenogamous species in the Gran Sabana Plateau. Therefore, breeding systems may explain, to some extent, the occurrence of endemism under current distribution and organization of herbaceous-shrubby communities. Previous studies have demonstrated the restricted distribution of endemic species (Lavergne *et al.* 2005) in an oceanic island (Bernardello *et al.* 2001). Accordingly, xenogamy and isolation among plant communities may be significant factors determining endemism in the herbaceous-shrubby communities in the Gran Sabana Plateau. In this context, self-incompatibility and partial self-incompatibility appear to be closely related with the speciation process in the heterogeneous mosaic of herbaceous-shrubby communities dispersed on the matrix of savanna and forest.

The highest frequency of partial self-incompatibility in the shrublands (Jardín) concurs with the high incidence of self-incompatibility in trees and liana and the high frequency of partially self-incompatible species in the secondary bushland may be also associated with the occurrence of herbaceous species in this community. Furthermore, partially self-incompatible species were higher among anemophilous than among biotic-pollinated species, and more than 50 % of plant species were oligophilous and polyphilous. Generalist pollination systems are commons in partially self-incompatible species from disturbed areas such as secondary bushland. In this context, partial self-incompatibility might be evolutionarily influenced under generalist pollination system.

Self-incompatible species were less frequent in the fallow and broad-leaved meadow and more frequent in the secondary bushland. Herbs are the most common life forms in the fallow and broad-leaved meadow (Ramírez *et al.* 2012), and self-compatibility has been reported as the main breeding system in herbaceous species (Lemus-Jiménez and Ramírez 2005). The frequency of self-incompatibility and partial self-incompatibility were found closely alike in the secondary bushland, which may be somehow determined by the relationship between plant life form and breeding system. The secondary bushland exhibited the highest life form diversity recorded in this area (Ramírez *et al.* 2012) what contribute to the diversity of breeding systems in this community. Specifically, the occurrence of self-incompatibility in woody species and partially self-incompatible in herbaceous species may, therefore, determine the frequency of the breeding system in the secondary bushland.

Cross-incompatibility is a breeding system category poorly examined at the community level. However, intra-population partial cross-incompatibility and total cross-incompatibility have been found in many species (Ramírez and Nassar 2016). In the current study, partially cross-incompatible species ranged from 23.3 to 30.8 % across plant communities and only one species was considered cross-incompatible in the plant communities examined. Partially cross-incompatible species was higher in the fallow primarily related to the predominance of herbaceous-colonizing species. In addition, the largest frequency of partially cross-incompatibility was found for monophilous species. High specificity of the pollination system may be unfavourable when pollinators are limited (Pérez *et al.* 2009) and may stimulate self-compatibility, and subsequently cross-incompatibility. Ecological circumstances also play an important role in determining when selfing evolves (Busch and Delph 2012). Cross-incompatibility has been found in a significant number of species in isolated or partially isolated communities, where pollinator activity is limited, and climate conditions are adverse (Ramírez and Nassar 2016). Therefore, isolation of shrublands and broad-leaved meadow together with deficiency of pollination service may be ecological factors stimulating cross-incompatibility in the herbaceous-shrubby communities of the Gran Sabana Plateau.

### Breeding system, sexuality, dichogamy and herkogamy

Sexual traits are variedly related with contrasting breeding systems. Herkogamy has been found in self-compatible and agamospermous species and non-herkogamous species in self-compatible, self-incompatible and agamospermous species (Hokche and Ramírez 2008). Likewise, partially constrained assisted self-pollination and partially endogamous were mainly hermaphrodite, herkogamous, adichogamous and dichogamous species, which suggest that obligate self-pollination and partial endogamy are not limited to plant species with rigid mechanisms that impose self-pollination. Thus, herkogamy and dichogamy have to be incomplete devices avoiding self-pollination in partially constrained assisted self-pollinated and partially endogamous species.

Self-incompatibility and partial self-incompatibility were mostly associated with hermaphroditism, herkogamy and adichogamy. Herkogamy represents the only sexual attribute that may avoid interference pollen–stigma and promotes cross-pollination in some partially self-incompatible and in many self-incompatible species. It was also found that hermaphrodite and monoecy occur in a similar frequency along with the categories of the self-incompatibility index, which suggests that self-fertility was independent of sexual systems, such as reported by Bertin (1993). Moreover, dichogamy has been found equally common among self-incompatible and self-compatible species (Bertin 1993), which is in concordance with the incidence of protandry in more than a few partially self-incompatible species studied and may be interpreted as an attribute promoting cross-pollination. In a similar way, partially cross-incompatible were mainly hermaphrodite, herkogamous and adichogamous species. These plant species may allow self-fertilization through hermaphroditism and adichogamy. Herkogamy may, in theory, avoid self-pollination; however, incomplete herkogamy might allow self-pollination. Indeed, some levels of cross-pollination occur in partially cross-incompatible species.

## Conclusions

The proportion of dioecious species in shrublands and secondary bushland is higher than communities primarily herbaceous where monoecious species dominate. The comparative abundance of dioecious species in shrublands and secondary bushland is mainly associated with shrubs and endemism, which may be phylogenetically influenced. The significant association between monoecy and herbaceous communities is phylogenetically influenced by the abundance of submonoecy in Poaceae and Cyperaceae, which are associated with the herbaceous life form, wind pollination and the C_4_ photosynthetic pathway. The frequency of herkogamy was approximately twice the frequency of dichogamy. The frequency of herkogamy is associated with vegetation structure and life form: high frequency of herkogamy for trees, shrubs and liana and the low values for herbaceous species. Non-herkogamy was higher where pollination systems, including wind pollination, became more generalist. The frequency of adichogamous was higher than dichogamous species. Protandry was significant in herbs, wind pollination and secondary vegetation, and protogyny may be high in contrasting vegetation types. Most plant species were non-agamospermous, non-spontaneous self-pollinated and xenogamous. Partial endogamy was higher in herbaceous species. Partial self-incompatibility dominated, followed by self-incompatibility, partial cross-incompatibility and cross-incompatibility in the sample examined. Self-incompatible species were more frequent in the secondary bushland. Sexual traits were found variedly related with contrasting breeding systems.

## Sources of Funding

This research was supported by BID-CONICIT, Autoridad Gran Sabana and Fundacite Guayana.

## Contributions by the Authors

N.R. and O.H. conceived and designed the study, collected the data, wrote the first draft, conducted the statistical analysis and N.R. led the revisions with major contributions. N.R. and O.H. edited and approved the final manuscript.

## Supporting Information

The following additional information is available in the online version of this article —

**Appendix 1.** Plant species, community, sexual system, temporal sexual expression and spatial sexual separation of 348 plant species of herbaceous-shrubby communities in the Gran Sabana Plateau.

**Appendix 2.** Results of experimental tests for 103 plant species from herbaceous-shrubby communities in the Gran Sabana Plateau.

**Appendix 3.** Breeding system indexes and their qualitative categories.

**Table S1.** Frequency of sexual system, temporal sexual expression, and spatial sexual separation according to some functional plant traits.

**Table S2.** Relationship between breeding systems and functional groups.

plz032_Suppl_Supplementary_Material
