# Supplementary material for: Outbreeding and inbreeding strategies in herbaceous-shrubby communities in the Venezuelan Gran Sabana Plateau
Source: AoB Plants. 2019 Jun 26;11(4):plz032. doi: 10.1093/aobpla/plz032 (PMC6621913; doi:10.1093/aobpla/plz032)
Supplement: plz032_Suppl_Supplementary_Material [file plz032_suppl_supplementary_material.pdf]

Table 1. Frequency of sexual system, temporal sexual expression, and spatial sexual separation according to some functional plant traits.

Functional group and plant communities	Sexuality			Temporal sexual expression			Spatial sexual separation	
	Hermaphrodite N(%)	Monoecy N(%)	Dioecy N(%)	Adichogamy N(%)	Protandry N(%)	Protogyny N(%)	Herkogamy N(%)	Non- Herkogamy N(%)
Life form	df = 8 χ^2 = 68.6(0.00000)			df = 8; χ^2 = 21.8(0.00536)			df = 4; χ^2= 31.1(0.00001)	
Trees	13(54.17)	3(12.50)	8(33.33)	11(68.75)	4(25.00)	1(6.25)	21(91.30)	2(8.70)
Shrubs	61(75.31)	4(4.94)	**16(19.75)**[a]	53(79.10)	9(13.43)	5(7.46)	68(93.15)	5(6.85)
Liana	18(78.26)	1(4.35)	4(17.39)	16(84.21)	3(15.79)	0(0.00)	22(100.00)	0(0.00)
Perennial Herbs	130(71.43)	48(26.37)	4(2.20)	92(52.54)	57(32.20)	27(15.25)	122(68.54)	56(31.46)
Annual Herbs	45(93.75)	3(6.25)	0(0.00)	30(63.83)	14(29.79)	3(6.38)	31(64.58)	17(35.42)
Succulence	df = 2; χ^2 = 31.1(0.00001)			df = 2; χ^2 = 1.3(n.s.)			df = 1; χ^2 = 0.47(n.s.)	
Non-Succulent	250(75.53)	56(16.92)	25(7.55)	191(62.42)	80(26.14)	35(11.44)	245(76.32)	76(23.68)
Succulent	17(62.96)	3(11,11)	7(25.93)	12(60.00)	7(35.00)	1(5.00)	19(82.61)	4(17.39)
Substrate Type	df = 2; χ^2 = 11.2(0.00365)			df = 2; χ^2; 1.6(n.s.)			df = 1; χ^2 = 2.1(n.s.)	
Terrestrial	262(75.94)	55(15.94)	28(8.12)	201(63.41)	81(25.55)	35(11.04)	253(76.20)	79(23.80)
Epiphytic	5(38.46)	4(30.77)	4(30.77)	2(22.22)	6(66.67)	1(11.11)	11(91.67)	1(8.33)
Nutritional relations	df = 4; χ^2 = 17.7(0.00143)			df = 4; χ^2 = 11.6(0.02018)			df = 2; χ^2 = 2.4(n.s.)	
Autotroph	249(75.00)	55(16.57)	28(8.43)	187(61.51)	82(26.97)	35(11.51)	245(76.80)	74(23.20)
Insectivore	14(100.00)	0(0.00)	0(0.00)	13(92.86)	0(0.00)	1(7.14)	9(64.29)	5(35.71)
Parasitic-Hemiparasitic	4(33.3)	4(33.3)	4(33.3)	3(37.50)	5(62.50)	0(0.00)	10(90.91)	1(9.09)
Carbon Metabolism	df = 4; χ^2 = 24.2(0.00007)			df = 4; χ^2 = 46.1(0.00000)			df = 2; χ^2 = 28.6(0.00001)	
C_3	242(77.07)	44(14.01)	28(8.92)	195(67.94)	65(22.65)	27(9.41)	245(80.59)	59(19.41)
C_4	18(54.55)	**15(45.45)**[b]	0(0.00)	**3(9.09)**[d]	**22(66.67)**[b]	8(24.24)	13(39.39)	**20(60.61)**[b]
CAM	6(85.71)	0(0.00)	1(14.29)	5(83.33)	0(0.00)	1(16.67)	6(85.71)	1(14.29)
Successional stage	df = 2; χ^2 = 9.2(0.01003)			df = 2; χ^2 = 2.9(n.s.)			df = 1; χ^2 = 0.1(n.s.)	
Late seral	142(71.00)	32(16.00)	26(13.00)	116(66.29)	43(24.57)	16(9.14)	147(76.96)	44(23.04)
Pioneer	125(79.11)	27(17.09)	6(3.80)	87(57.62)	44(29.14)	20(13.25)	117(76.47)	36(23.53)
Pollination System	df = 6; χ^2 = 56.5(0.00000)			df = 6; χ^2 = 76.5(0.00000)			df = 3; χ^2 = 40.0(0.00000)	
Monophily	65(83.33)	6(7.69)	7(8.97)	58(80.56)	8(11.11)	6(8.33)	69(88.46)	9(11.54)
Oligophily	39(92.86)	**0(0.0)**[d]	3(7.14)	30(76.92)	8(20.51)	1(2.56)	36(87.80)	5(12.20)
Polyphily	44(73.33)	8(13.33)	8(13.33)	30(57.69)	17(32.69)	5(9.62)	44(74.58)	15(25.42)
Anemophily	25(46.30)	**27(50.00)**[b]	2(3.70)	**4(7.69)**[d]	**32(61.54)**[b]	**16(30.77)**[a]	22(42.31)	**30(57.69)**[b]
Endemism	df = 2; χ^2 = 9.9(0.00685)			df = 2; χ^2 = 1.6(n.s.)			df = 1; χ^2 = 2.1(n.s.)	
Non-Endemic	253(75.30)	57(16.96)	26(7.74)	190(61.49)	84(27.18)	35(11.33)	252(77.54)	73(22.46)
Endemic	14(63.64)	2(9.09)	**6(27.27)**[a]	13(76.47)	3(17.65)	1(5.88)	12(63.16)	7(36.84)
Community	df = 12; χ^2 = 20.46(n.s.)			df = 12; χ^2 = 22.4(0.03274)			df = 6; χ^2 = 25.7(0.00025)	
Shrubland (Jardin)	85(75.89)	16(14.29)	11(9.82)	68(66.67)	22(21.57)	12(11.76)	89(81.65)	20(18.35)
Shrubland (Liworiwo)	93(76.23)	17(13.93)	12(9.84)	77(70.00)	24(21.82)	9(8.18)	98(83.05)	20(16.95)
Shrubland (Mareman)	105(72.41)	21(14.48)	19(13.10)	81(63.78)	32(25.20)	14(11.02)	114(82.01)	25(17.99)
Secondary bushland	104(76.47)	21(15.44)	11(8.09)	76(61.29)	37(29.84)	11(8.87)	97(73.48)	35(26.52)
Broad-leaved meadow	85(80.19)	17(16.04)	4(3.77)	66(64.71)	27(26.47)	9(8.82)	76(72.38)	29(27.62)
Savanna	74(76.29)	21(21.65)	2(2.06)	46(48.94)	35(37.23)	13(13.83)	57(60.00)	38(40.00)
Fallow	41(74.55)	13(23.64)	1(1.82)	81(63.78)	32(25.20)	14(11.02)	34(64.15)	19(35.85)

Values in bold face indicate significant residual and superscript indicates: [a] = Positive Residual at P<0.05; [b] = Positive Residual at P<0.01; [d] = Negative Residual at P<0.01

Table 2. Relationship between breeding systems and functional groups.

Functional group and communities	Breeding System Indexes												
	Agamospermy (IAG)			Spontaneous self-pollination (ISSP)			Self-fertility (ISF)			Self-incompatibility (ISI)			
	Categories[1]			Categories[2]			Categories[3]			Categories[4]			
	NAG	PAG	PCSM	NSSP	PSSP	PCASP	X	PX	PE	SI	PSI	PCI	CI
	N(%)	N(%)	N(%)	N(%)	N(%)	N(%)	N(%)	N(%)	N(%)	N(%)	N(%)	N(%)	N(%)
Life form		**			**			**			**		
Trees	6(75.0)	2(25.0)	0(0.0)	6(75.0)	2(25.0)	0(0.0)	5(100.0)	0(0.0)	0(0.0)	4(80.0)	1(20.0)	0(0.0)	0(0.0)
Shrubs	29(76.3)	8(21.1)	1(2.6)	23(63.9)	8(22.2)	5(13.9)	26(76.5)	6(17.6)	2(5.9)	12(37.5)	11(34.4)	8(25.0)	1(3.1)
Liana	5(100.0)	0(0.0)	0(0.0)	8(100.0)	0(0.0)	0(0.0)	10(100.0)	0(0.0)	0(0.0)	6(85.7)	0(0.0)	1(14.3)	0(0.0)
Perennial Herbs	31(83.8)	5(13.5)	1(2.7)	34(64.2)	15(28.3)	4(7.5)	34(64.2)	12(22.6)	7(13.2)	5(14.3)	22(62.9)	8(22.9)	0(0.0)
Annual Herbs	3(75.0)	1(25.0)	0(0.0)	1(16.7)	3(50.0)	2(33.3)	1(25.0)	1(25.0)	2(50.0)	0(0.0)	2(66.7)	1(33.3)	0(0.0)
Succulence		***df=1; χ^2 = 1.37(n.s.)			df=2; χ^2 = 3.27(n.s.)			df=2; χ^2 = 3.23(n.s.)			**		
Non-Succulent	69(80.2)	16(18.6)	1(1.2)	60(61.9)	27(27.8)	10(10.3)	65(69.2)	19(20.2)	10(10.6)	27(35.0)	33(42.9)	16(20.8)	1(1.3)
Succulent	6(85.7)	0(0.0)	1(14.3)	12(85.8)	1(7.1)	1(7.1)	11(91.7)	0(0.0)	1(8.3)	0(0.0)	3(60.0)	2(40.0)	0(0.0)
Substrate Type		df=1; χ^2 = 0.66(n.s.)			df=1; χ^2 = 0.09(n.s.)			df=1; χ^2 =0.04(n.s.)			**		
Terrestrial	72(80.0)	16(17.8)	2(2.2)	67(64.4)	26(25.0)	11(10.6)	71(71.0)	18(18.0)	11(11.0)	27(33.7)	34(42.5)	18(22.5)	1(1.3)
Ephiphytic	3(100.0)	0(0.0)	0(0.0)	5(71.4)	2(28.6)	0(0.0)	5(83.3)	1(16.7)	0(0.0)	0(0.0)	2(100.0)	0(0.0)	0(0.0)
Nutritional relations		**			**			**			**		
Autotroph	74(82.2)	14(15.6)	2(2.2)	67(64.4)	27(26.0)	10(9.6)	71(71.7)	18(18.2)	10(10.1)	27(34.2)	33(41.8)	18(22.8)	1(1.3)
Insectivore	0(0.0)	1(100.0)	0(0.0)	0(0.0)	0(0.0)	1(100.0)	0(0.0)	0(0.0)	1(100.0)	0(0.0)	1(100.0)	0(0.0)	0(0.0)
Parasitic-Hemiparasitic	1(50.0)	1(50.0)	0(0.0)	5(83.3)	1(16.7)	0(0.0)	5(83.3)	1(16.7)	0(0.0)	0(0.0)	2(100.0)	0(0.0)	0(0.0)
Carbon Metabolism		**			**			**			**		
C^3	65(78.3)	16(19.3)	2(2.4)	64(64.0)	26(26.0)	10(10.0)	68(70.8)	17(17.7)	11(11.5)	26(35.1)	30(40.5)	17(23.0)	1(1.4)
C^4	5(100.0)	0(0.0)	0(0.0)	3(60.0)	1(20.0)	1(20.0)	4(66.7)	2(33.3)	0(0.0)	1(20.0)	4(80.0)	0(0.0)	0(0.0)
CAM	5(100.0)	0(0.0)	0(0.0)	5(83.3)	1(16.7)	0(0.0)	4(100.0)	0(0.0)	0(0.0)	0(0.0)	2(66.7)	1(33.3)	0(0.0)
Successional stage		***df=1; χ^2 = 2.47(n.s.)			***df=1; χ^2 = 0.13(n.s.)			***df=1; χ^2 = 1.51(n.s.)			***df=1; χ^2 = 1.63(n.s.)		
Late seral	48(84.2)	7(12.3)	2(3.5)	46(68.7)	17(25.4)	4(5.9)	47(71.2)	14(21.2)	5(7.6)	17(32.7)	24(46.2)	10(19.2)	1(1.9)
Pioneer	26(74.3)	9(25.7)	0(0.0)	27(61.4)	11(25.0)	6(13.6)	30(75.0)	4(10.0)	6(15.0)	10(33.3)	12(40.0)	8(26.7)	0(0.0)
Pollination Systems		***df=3; χ^2 = 2.47(n.s.)			***df=3; χ^2 = 4.98(n.s.)			***df=3; χ^2 = 4.8(n.s.)			***df=6; χ^2 =10.78(n.s.)		
Monophily	23(76.7)	6(20.0)	1(3.3)	25(69.4)	9(25.0)	2(5.6)	25(78.1)	5(15.6)	2(6.3)	7(30.4)	6(26.1)	9(39.1)	1(4.4)
Oligophily	17(77.3)	5(22.7)	0(0.0)	14(66.7)	5(23.8)	2(9.5)	13(61.9)	6(28.6)	2(9.5)	5(25.0)	11(55.0)	4(20.0)	0(0.0)
Polyphily	18(81.8)	3(13.6)	1(4.6)	13(59.1)	5(22.7)	4(18.2)	16(69.6)	4(17.4)	3(13.0)	8(42.1)	9(47.4)	2(10.5)	0(0.0)
Anemophily	8(100.0)	0(0.0)	0(0.0)	3(33.3)	5(55.6)	1(11.1)	5(50.0)	4(40.0)	1(10.0)	1(12.5)	6(75.0)	1(12.5)	0(0.0)
Endemism		**			**			**			**		
Non-Endemic	70(79.5)	16(18.2)	2(2.3)	66(63.5)	27(25.9)	11(10.6)	71(71.0)	18(18.0)	11(11.0)	26(33.3)	34(43.6)	17(21.8)	1(1.28)
Endemic	5(100.0)	0(0.0)	0(0.0)	6(85.7)	1(14.3)	0(0.0)	5(83.3)	1(16.7)	0(0.0)	1(25.0)	2(50.0)	1(25.0)	0(0.0)
Community		***df= 6; χ^2 = 4.22(n.s.)			df=12; χ^2 = 3.36(n.s.)			df=12; χ^2 = 5.39(n.s.)			*** df= 12; χ^2 = 4.54(n.s.)		
Shrubland (Jardin)	38(79.2)	8(16.7)	2(4.1)	33(63.5)	13(25.0)	6(11.5)	37(69.8)	9(17.0)	7(13.2)	8(18.6)	24(55.8)	10(23.3)	1(2.3)
Shrubland (Liworiwo)	36(78.3)	8(17.4)	2(4.3)	33(63.5)	13(25.0)	6(11.5)	35(70.0)	10(20.0)	5(10.0)	10(24.4)	21(51.2)	10(24.4)	0(0.0)
Shrubland (Mareman)	43(79.6)	10(18.5)	1(1.9)	40(64.5)	14(22.6)	8(12.9)	42(71.2)	12(20.3)	5(8.5)	12(25.5)	23(49.0)	12(25.5)	0(0.0)
Secondary bushland	23(74.2)	8(25.8)	0(0.0)	24(60.0)	12(30.0)	4(10.0)	27(73.0)	8(21.6)	2(5.4)	10(35.7)	11(39.2)	7(25.0)	0(0.0)
Broad-leaved meadow	30(78.9)	6(15.8)	2(5.3)	27(62.8)	12(27.9)	4(9.3)	29(64.4)	8(17.8)	8(17.8)	7(18.9)	19(51.4)	10(27.0)	1(2.7)
Savanna	25(92.6)	2(7.4)	0(0.0)	18(58.1)	8(25.8)	5(16.1)	22(66.7)	6(18.2)	5(15.1)	8(28.6)	13(46.4)	7(25.0)	0(0.0)
Fallow	11(91.7)	1(8.3)	0(0.0)	7(46.7)	5(33.3)	3(20.0)	8(57.1)	4(28.6)	2(14.3)	2(15.4)	7(53.8)	4(30.8)	0(0.0)

[1] Agamospermy index categories: NAG = non-agamospermous, PAG = partially agamospermous, PCSM = partially constrained sexual mating. [2] Spontaneous self-pollination index categories: NSSP = non-spontaneous self-pollinated, PSSP = partially spontaneous self-pollinated, PCASP = partially constrained assisted self-pollination. [3] Self-fertility index categories: X = xenogamous, PX = partially xenogamous, PE = partially endogamous. [4] Self-incompatibility index categories: SI = self-incompatible, PSI = partially self-incompatible, PCI = partially cross-incompatible, CI = cross-incompatible. ** = Statistical analysis was not performed because data set do not fit to Chi square test; *** = these results were performed excluding PCSM; PCASP; PE and CI species, respectively.

Appendix 1. Plant species, community, sexual system, temporal sexual expression, and spatial sexual separation of 348 plant species of herbaceous-shrubby communities in the Gran Sabana Plateau.

Family Species	Community[a]	Habitat[b]	Sexuality[c]	Temporal sexual expression[d]	Spatial sexual separation[e]
**ACANTHACEAE**					
Justicia guianensis (N.E. Br.) Wassh.	R	D	H	ADIC	NH
**ANACARDIACEAE**					
Anacardium occidentale L.	R	D	H	PT	H
**APOCYNACEAE**					
Blepharodon pictum (Vahl) W.D. Stevens	H;R;S	D	H	ADIC	H
B. ulei Schltr.	H;L;R	D	H	ADIC	H
Ditassa bolivarensis (R.W. Holm) Morillo	R;S	M	H	ADIC	H
D. tatei Gleason & Moldenke	H;L;R	M	H	ADIC	H
Galactophora schomburgkiana Woodson var. *schomburgkiana*	J;L	M	H	ADIC	NH
Mandevilla benthamii (A. DC.) K. Schum.	H;J;L;M;R;S	M	H	ADIC	H
M. gracilis (Kunth) J.F. Morales	L;R	D	H	ADIC	H
Metastelma hirtella (Oliv.) Liede	M;R;S	D	H	ADIC	H
**AQUIFOLIACEAE**					
Ilex danielis Killip & Cuatrec.	M;R	M	D[(hh)]	-	H
I. polita Steyerm.	J;L	M	D[(hh)]	-	H
I. subrotundifolia Steyerm.	J	M	D[(hh)]	-	NH
I. retusa Klotzsch	J;L:M	M	D[(hh)]	-	NH
**ARACEAE**					
Philodendron ptarianun Steyerm. var. *rugosum*	J;L:M	M	M	PG	H
**ASTERACEAE**					
Achyrocline satureioides (Lam.) DC.	R;F	D	GM	PT	NH
Ageratum conyzoides L. subsp. *latifolium* (Cav.) M.F. Johnson	R	D	H	PT	NH
Austroeupatorium inulifolium (Kunth) R.M. King & H. Rob.	F;R	D	GM	PT	NH
Ayapana amygdalina (Lam.) R.M. King & H. Rob.	F;R;S	D	H	ADIC	NH
Baccharis leptocephala DC.	F;R;S	D	GD	-	NH
Bidens pilosa L.	R	D	H	ADIC	NH
Calea alchioides S.F. Blake	R	D	GM	PG	NH

C. cardonae Maguire & Wurdack	L	M	GM	PG	NH
C. divaricata Benth.	M	M	GM	PG	NH
C. ludivenia Gleason & S.F. Blake	H;J;L;M	M	GM	PG	NH
C. nana Maguire	S	M	GM	PG	NH
C. oliveri B.L. Rob. & Greenm.	S	D	GM	PG	H
Centratherum punctatum Cass. var. *punctatum*	R	D	H	PG	H
Chaptalia integerrima (Vell.) Burkart	F	D	GM	PG	H
Chromolaena laevigata (Lam.) R.M. King & H. Rob.	F;J;M;R;S	D	H	PT	H
C. thurnii (B.L. Rob.) R.M. King & H. Rob.	F;J;S	D	H	PT	NH
Conyza bonariensis (L.) Cronquist	F	D	GM	PG	NH
Elephantopus mollis Kunth	F	D	H	PT	NH
Erechtites hieracifolius (L.) Raf. ex DC.	R	D	H	PT	NH
Gongylolepis benthamiana R.H. Schomb.	J;L;M	M	H	PT	H
Ichthyothere terminalis (Spreng.) S.F. Blake	R	D	GM	PT	H
Lepidaploa bolivarensis (V.M. Badillo) H. Rob.	J;M	D	H	PT	H
L. ehretiifolia (Benth.) H. Rob.	S	D	H	PT	H
L. gracilis (Kunth) H. Rob.	R;S	D	H	PT	H
L. salzmannii (DC.) H. Rob.	F;R;S	D	H	ADIC	H
Mikania micrantha Kunth	R	D	H	PT	H
M. psilostachya DC.	J;R	D	H	PT	H
Stomatochaeta condensata (Baker) Maguire & Wurdack	H;J;L;M	M	H	PT	H
Trichogonia campestris Gardner	F;R;S	D	H	ADIC	NH
**BIGNONIACEAE**					
Digomphia laurifolia Benth.	H;J;L;M	M	H	ADIC	H
**BONNETIACEAE**					
Bonnetia sessilis Benth.	H;J;L;M	M	H	ADIC	H
**BROMELIACEAE**					
Brocchinia acuminata L.B. Sm.	J	M	H	ADIC	NH
B. reducta Baker	H;J;LM	M	H	ADIC	NH
B. steyermarkii L.B. Sm.	H	M	H	PT	NH
Catopsis berteroniana (Schult. & Schult. f.) Mez	L;M	M	H	PT	NH
Lindmania geniculata L.B. Sm. var. *geniculata*	M	M	H	PT	NH
L. guianensis (Beer) Mez	H;J;L;M	M	H	PT	NH
Pitcairnia brittoniana Mez	R	M	H	ADIC	NH

Tillandsia flexuosa Sw.	L	M	H	PG	H
**BURMANNIACEAE**					
Burmannia bicolor Mart.	H;M	M	H	ADIC	NH
**BURSERACEAE**					
Dacryodes roraimensis Cuatrec.	L;M;R	M	D(hh)	-	H
**CALOPHYLLACEAE**					
Mahurea exstipulata Benth.	R	M	H	ADIC	H
**CAMPANULACEAE**					
Centropogon cornutus (L.) Druce	R	D	H	PT	H
Lobelia fastigiata Kunth	S	D	H	PT	H
**CHRYSOBALANACEAE**					
Hirtella scabra Benth.	L	M	H	ADIC	H
**CLUSIACEAE**					
Clusia (grupo sessilis) sp.	L	M	D(ad)	-	H
Clusia aff. brachystyla Maguire	M	M	D(ad)	-	H
C. amabilis Maguire	L	M	D(ad)	-	H
C. columnaris Engl.	R	M	D(ad)	-	H
C. crassifolia Planch. & Triana	H;M	M	D(ad)	-	H
C. fockeana Miq.	L;M	M	D(ad)	-	H
C. grandiflora Splitg.	J;R	D	D(ad)	-	H
C. huberi Pipoly	M	M	D(ad)	-	H
C. pusilla Steyerm. ssp. *pusilla*	H;J;M	M	D(ad)	-	H
C. schomburgkiana (Planch. & Triana) Benth. ex Engl.	M;R	M	D(hh)	-	H
**CYPERACEAE**					
Bulbostylis conifera (Kunth) C.B.Clarke	S	M	AM	PG	H
B. juncoides (Vahl) Kük. ex Osten	F	D	H	PG	H
B. junformis (Kunth) C.B. Clarke	J;L;M	D	H	PG	H
B. lanata (Kunth) Lindm.	F;M;S	D	H	PG	H
B. paradoxa (Spreng.) Lindm. (female)	S	M	AM	PT	H
Cephalocarpus rigidus Gilly	J;M	M	M	PT	H
Cyperus aggregatus (Willd.) Endl.	R	D	H	ADIC	H
Hypolytrum longifolium (L.C. Rich.) Nees ssp. *nicaraguense* (Liebm.) T. Koyama	H	D	H	ADIC	H

H. pulchrum (Rudge) H. Pfeiff.	F;H;M;R;S	M	H	PT	H
Lagenocarpus rigidus Kunth (Ness)	F;H;J;L;M;R;S	M	M	PT	H
Mapania tepuiana (Steyerm.) T. Koyama	H;L;M	M	M	PT	H
Rhynchospora barbata (Vahl.) Kunth	F;H;J;L;M;R;S	D	AM	PT	NH
R. caracasana (Kunth) Boeckeler (male)	J;L;M;S	M	AM	PG	NH
R. cephalotes (L.) Vahl	R	M	AM	PG	NH
R. curvula Griseb.	S	M	H	PG	H
R. exaltata Kunth	J	D	AM	PG	H
R. filiformis Vahl	H	M	H	PT	NH
R. globosa (Kunth) Roem. & Schult.	H;M;S	M	AM	PG	H
R. marisculus Nees	H	M	AM	PG	H
R. mexicana (Liebm.) Steud.	S	M	AM	ADIC	H
R. pilosa (Kunth) Boeck.	H;J;L;M;S	D	AM	PG	H
R. rugosa (Vahl) Gale	F;S	D	H	PG	H
R. tenuis Willd. ex Link	M;F	D	H	PG	H
R. velutina (Kunth) Boeck.	S	D	AM	PG	NH
Scleria cyperina Willd. ex Kunth	F;H;J;M;R;S	M	M	PG	H
S. distans Poir.	S	D	M	PG	H
**CYRILLACEAE**					
Cyrilla racemiflora L.	H;J;L;M	M	H	ADIC	H
**DROSERACEAE**					
Drosera arenicola Steyerm.	H	M	H	ADIC	NH
D. felix Steyerm. & L.B. Sm.	H;J;L;M;S	M	H	ADIC	NH
D. roraimae (Klotzsch ex Diels) Maguire & J.R. Laundon	H	M	H	ADIC	NH
**ERICACEAE**					
Bejaria sprucei Meisn.	L;M;R	M	H	ADIC	H
Notopora schomburgkii Hook. f.	L;M;R	M	H	ADIC	H
Orthaea merumensis Maguire, Steyerm. & Luteyn	M	M	H	ADIC	H
Vaccinium euryanthum A.C. Sm.	J;L;M	M	H	ADIC	H
V. puberulum Klotzsch ex Meisn.	J;L;M	M	H	ADIC	H
**ERIOCAULACEAE**					
Leiothrix flavescens (Bong.) Ruhland (female)	H	M	M	PG	H
Paepalanthus dichotomus Klotzsch ex Körn.	H;L;M	M	M	PT	H

Syngonanthus aff. simplex (Miq.) Ruhland	H	M	M	PT	H
S. gracilis (Bong.) Ruhland	J	M	M	PT	H
S. longipes Gleason	H	M	M	PT	H
S. pakaraimensis Mold. var. *pakaraimensis* (female)	J;L;M	M	M	PT	H
S. xeranthemoides (Bong.) Ruhland	J;H;L;M	M	M	PT	H
**ERYTHROXYLACEAE**					
Erythroxylum trifolium A. St.-Hil.	R	D	H[(d)]	ADIC	H
**EUPHORBIACEAE**					
Chaetocarpus schomburgkianus (Kuntze) Pax & K. Hoffm.	M;R	D	AGD	-	H
**EUPHRONIACEAE**					
Euphronia guianensis (R.H. Schomb.) Hallier f.	J;L;M	M	H	PT	H
**FABACEAE**					
Abarema ferruginea (Benth.) Pittier	M	M	AM	ADIC	NH
A. jupunba (Willd.) Britton & Killip	R	D	AM	ADIC	H
Calliandra pakaraimensis R.S. Cowan	L	M	AM	ADIC	H
Chamaecrista desvauxii (Benth.) H.S. Irwin & Barneby	L;M;S	M	H	ADIC	H
C. ramosa (Vogel) H.S. Irwin & Barneby	L	M	H	ADIC	H
Dalbergia monetaria L. f.	R	M	H	ADIC	H
Dicymbe fraterna Cowan	L	M	H	ADIC	H
Dimorphandra macrostachya Benth. ssp. macrostachya	R	M	H	ADIC	H
Mimosa pudica L. var. *tetrandra* (Humb. & Bonpl. ex Willd.) DC.	F	D	AM	ADIC	H
Taralea cordata Ducke	L;M	D	H	ADIC	H
**GENTIANACEAE**					
Chelonanthus angustifolius (Kunth) Gilg.	H;L;M;S	D	H	PT	H
C. purpurascens (Aubl.) Struwe & V.A. Albert	F;M;R	D	H	PT	H
Coutoubea reflexa Benth.	H;M;S	D	H	PT	NH
Curtia tenuifolia (Aubl.) Knobl. ssp. *tenuifolia*	H;L;R;S	M	H	ADIC	NH
Irlbachia nemorosa (Willd. ex Roem. & Schult.) Merr.	H;J;M	D	H	ADIC	H
Tetrapollinia caerulescens (Aubl.) Maguire & B.M. Boom	H;L;M	D	H	PT	H
**GNETACEAE**					
Gnetum camporum (Markgr.) D.W. Stev. & Zanoni	M	D	D[(u)**]	-	H

**HUMIRIACEAE**					
Humiria balsamifera Aubl.	H;J;L;M	M	H	ADIC	NH
Vantanea minor Benth.	L	M	H	PT	H
**HYPERICACEAE**					
Vismia guianensis (Aubl.) Choisy	J;R;S	D	H	ADIC	H
**IRIDACEAE**					
Sisyrinchium vaginatum Spreng.	R	D	H	ADIC	H
Trimezia fosteriana Steyerm.	H;J	M	H	PT	H
**IXONANTHACEAE**					
Ochthocosmus attenuatus Steyerm. & Luteyn	M	M	H	PT	H
O. longipedicellathus Steyerm. & Luteyn	H;L;M	M	H	PT	H
O. roraimae Benth. var. *roraimae*	L	M	H	ADIC	H
**LAMIACEAE**					
Aegiphila integrifolia (Jacq.) Jacq. Ex B.D. Jacks. var. *guianensis* (Moldenke) López-Pal.	R	D	D[(d)]	-	H
Hyptis luticola Epling	R	D	H	PT	H
H. suaveolens (L.) Poit.	F	D	H	PT	H
**LAURACEAE**					
Cassytha filiformis L.	H;L;M	M	H	ADIC	H
Ocotea duidensis Moldenke	J	M	D[(hh)]	-	H
**LENTIBULAREACEAE**					
Genlisea filiformis A. St.-Hil.	M	M	H	ADIC	H
G. repens Benj.	H	M	H	ADIC	H
Utricularia hispida Lam.	H	M	H	ADIC	H
U. tenuissima Tutin	L	M	H	ADIC	H
U. amethystina St. Hil.	S	D	H	ADIC	H
U. juncea Vahl	H;L;M	M	H	ADIC	H
U. longeliata DC.	H;L;M	M	H	ADIC	H
U. subulata L.	L;M	M	H	ADIC	H
**LOGANIACEAE**					
Bonyunia minor N.E. Br.	L;M;S	M	H	PT	H
**LORANTHACEAE**					
Phthirusa stelis (L.) Kuijt	J;L;M;R	M	D[(ad)]	-	H
Struthanthus gracilis (Gleason) Steyerm. & Maguire	M;R	M	H	ADIC	H

Struthanthus sp.	L	M	D(ad)	-	H
S. syringifolius (Mart.) Eichler	M;R	M	D(ad)	-	H
Tripodanthus acutifolius (Ruiz & Pav.) Tiegh.	L	M	H	PT	H
**MALPHIGHIACEAE**					
Banisteriopsis martiniana (A. Juss.) Cuatrec. var. *martiniana*	R	D	H	ADIC	H
Byrsonima verbascifolia (L.) DC.	F;M;S	D	H	ADIC	H
Tetrapterys pusilla Steyerm.	J;L	M	H	ADIC	H
T. rhodopteron Oliv.	R	D	H	ADIC	H
Banisteriopsis pulcherrima (Sandwith) B. Gates	R	D	H	ADIC	H
Byrsonima concinna Benth.	M;R	D	H	ADIC	H
B. crassifolia (L.) Kunth	J;M;R;S	M	H	ADIC	H
Tetrapteris styloptera A. Juss.	R	D	H	PT	H
T. pusilla Steyerm.	H;J;L	M	H	ADIC	H
**MALVACEAE**					
Pachira aquatica Aubl.	R	M	H	ADIC	H
Sida linifolia Juss. ex Cav.	F	D	H	ADIC	H
Waltheria indica L.	F	D	H	ADIC	H
**MELASTOMATACEAE**					
Acotis acuminifolia (Mart. ex DC.) Triana	R	D	H	ADIC	H
Clidemia capitata Benth.	J;L;M	D	H	ADIC	H
C. capitellata (Bonpl.) D. Don	F;J;R	D	H	ADIC	H
C. octona (Bonpl.) L.O. Williams ssp. *guayanensis* Wurdack	R	D	H	ADIC	H
C. pustulata DC.	M;R;S	D	H	ADIC	H
C. pycnaster Tutin ssp. *pycnaster*	H;L;M	D	H	ADIC	H
C. sericea D. Don	F;M;R;S	D	H	ADIC	H
Comolia microphylla Benth.	H;J;L;M;R	M	H	ADIC	H
Desmoscelis villosa (Aubl.) Naudin	S	M	H	ADIC	H
Henriettea granulata O. Berg & Triana	R	D	H	ADIC	H
Macairea lasiophylla (Benth.) Wurdack	H;M;S	M	H	ADIC	H
M. pachyphylla Benth.	J	M	H	ADIC	H
M. parvifolia Benth.	J;L	M	H	ADIC	H
Marcetia taxifolia (A. St.-Hil.) DC.	F;H;J;L;M;R;S	D	H	PG	H
Meriania sclerophylla Triana	H;J;M	D	H	ADIC	H

M. urceolata Triana	H;M	D	H	ADIC	H
Miconia alata (Aubl.) DC.	F;R;S	D	H	PT	H
M. albicans (Sw.) Steud.	F;R	D	H	PG	H
M. aplostachya (Bonpl.) DC.	R	D	H	PG	H
M. ciliata (Rich.) DC.	H;J;L;M;R	D	H	ADIC	H
M. ibaguensis (Bonpl.) Triana	R	D	H	PG	H
M. phaeophylla Triana	J;M;R	D	H	PG	H
M. rubiginosa (Bonpl.) DC.	M;R	D	H	ADIC	H
M. rufescens (Aubl.) DC.	F:R;S	D	H	PT	H
M. stenostachya DC.	F;M;R	D	H	ADIC	H
Microlicia benthamiana Triana & Cogn.	H;J;L	M	H	PT	H
Siphanthera cordifolia (Benth.) Gleason	F;H;J;L;M;R;S	D	H	ADIC	H
Tibouchina fraterna N.E. Br. ssp. *fraterna*	H;J;L;M	D	H	ADIC	H
Tococa guianensis Aubl.	J;M;R	D	H	ADIC	H
T. nitens (Benth.) Triana	H;J;L;M	D	H	ADIC	H
**MYRTACEAE**					
Calycolpus goetheanus (DC.) O. Berg	R	D	H	ADIC	H
Eugenia protenta McVaugh	R	D	H	ADIC	H
E. punifolia (Kunth) DC.	R	D	H	ADIC	H
Myrcia albidotomentosa (Amshoff) McVaugh	J;L;M	M	H	ADIC	H
M. magnoliifolia DC.	R	D	H	ADIC	H
M. sylvatica (G. Mey.) DC.	L;M;R	D	H	ADIC	H
Psidium guineense Sw.	F;R	D	H	ADIC	H
P. laruotteanum Cambess.	S	M	H	ADIC	H
**NARTHEACEAE**					
Nietneria paniculata Steyerm.	H;L	M	H	ADIC	H
**OCHNACEAE**					
Ouratea gillejana (Dwyer) Sandwith & Maguire	J;L;M	M	H	ADIC	H
Poecilandra pumila Steyerm.	H;L	M	H	ADIC	H
P. retusa Tul.	J;L;M	M	H	ADIC	H
Sauvagesia amoena Ule	S	M	H	ADIC	H
S. angustifolia Ule	J;L	M	H	ADIC	H
S. erecta L. ssp. *erecta* var. *erecta*	F;R	D	H	ADIC	H
S. fruticosa Mart. & Zucc.	J;M	D	H	ADIC	H

S. guianensis (Eichler) Sastre	H	M	H	ADIC	H
S. rubiginosa A. St.-Hil.	L	M	H	ADIC	H
**ONAGRACEAE**					
Ludwigia erecta (L.) Hara	R	D	H	ADIC	NH
L. octovalvis (Jacq.) Raven	R	D	H	ADIC	NH
**ORCHIDACEAE**					
Catasetum discolor (Lindl.) Lindl.	H;J;L;M	M	D[u]	-	-
Cleistes moritzii (Rchb. f.) Garay & Dunst.	L;M;R	M	H	ADIC	H
C. rosea Lindl.	H;L;M;S	M	H	ADIC	H
C. stricta (C. Schweinf.) Garay & Dunst.	J	M	H	ADIC	H
C. unifoliata (C. Schweinf.) Carnevali & Ramírez	H	M	H	ADIC	H
Cyrtopodium parviflorum Lindl.	S	M	H	ADIC	H
Echinosepala arenicola (Carnevali & I. Ramírez) Carnevali & G. Romero	J	M	H	ADIC	H
Epidendrum dendrobioides Thunb.	H	M	H	ADIC	H
E. ibaguense Kunth	H;J:M;R;S	D	H	ADIC	H
E. orchidiflorum Salzm. ex Lindl.	J;L;M	M	H	ADIC	H
E. secundum Jacq.	F;H;J;L:M:S	D	H	ADIC	H
E. tumuc-humaciense (Veyret) Carnevali & G. Romero	H;J;L;M	M	H	ADIC	H
Epistephium duckei Huber	J;L;M	M	H	ADIC	H
E. subrepens Hoehne	H	M	H	ADIC	H
Eriopsis biloba Lindl.	H;J;L;M	M	H	ADIC	H
Habenaria mesodactyla Griseb.	H;L;M;R;S	M	H	ADIC	H
H. schomburgkii Lindl.	H;M;S	M	H	ADIC	H
Koellensteinia tricolor (Lindl.) Rchb. f.	H;J	M	H	ADIC	H
Maxillaria auyantepuiensis Foldats	J	M	H	ADIC	H
Scaphyglottis bidentata (Lindl.) Dressler	L;M	M	H	ADIC	H
Sobralia elisabethae R.H. Schomb.	J;L;M	M	H	ADIC	H
**OROBANCHACEAE**					
Buchnera palustris (Aubl.) Spreng.	H;L;M;R;S	D	H	ADIC	H
**PASSIFLORACEAE**					
Passiflora auriculata Kunth	F;J;R	D	H	ADIC	H
P. misera Kunth	R	D	H	ADIC	H
P. sclerophylla Harms	H;J;M	M	H	PT	H

**PENTAPHYLACACEAE**					
Ternstroemia crassifolia Benth.	J;L;M	M	AM	ADIC	H
T. pungens Gleason	J;L;M	M	H	ADIC	H
T. retusifolia Kobuski	L;M	M	H	ADIC	H
**PHYLLANTHACEAE**					
Phyllanthus majus Steyerm.	H;J	M	M	PG	H
P. stipulatus (Raf.) G.L. Webster	F;R	D	M	PG	H
**PLANTAGINACEAE**					
Scoparia dulcis L.	R	D	H	ADIC	H
**POACEAE**					
Andropogon bicornis L.	F;R	D	AM	PT	NH
A. selloanus (Hack.) Hack.	S	M	AM	PT	H
Aristida recurvata Kunth	F;H;S	M	H	PT	NH
A. torta (Nees) Kunth	R;S	M	H	PT	NH
Axonopus anceps (Mez) Hitchc.	F;H;J;L;M;R;S	M	H	PT	NH
A. canescens (Nees ex Trin.) Pilg.	R;S	M	H	PT	NH
A. fissifolius (Raddi) Kuhlm	S	D	H	PT	NH
A. flabelliformes Swallen	H;L;M;R;S	M	H	PT	NH
Axonopus sp. C (Flora of the Venezuelan Guayana, Vol. 8)	S	M	H	PT	NH
Dichanthelium sphaerocarpon (Elliot) Gould	J	M	H	PT	NH
Echinolaena inflexa (Poir) Chase	F;H;J;L;M;R;S	D	AM	PT	NH
Mesosetum rottboellioides (Kunth) Hitchc.	H;S	D	H	PT	NH
Panicum chnoodes Trin.	L	D	GM	PT	NH
P. cyanescens Nees ex Trin.	F;H;M;R;S	D	GM	PT	NH
P. micranthum Kunth	R;S	D	GM	PT	NH
P. nervosum Lam.	J;S	M	H	PT	NH
P. pilosum Sw.	R	M	H	PT	NH
P. rudgei Roem. & Schult.	F;R	D	H	PT	NH
P. stenodes Griseb.	S	D	H	PT	NH
P. lanciflorum Trin.	F;H;M;S	D	H	ADIC	NH
Paspalum stellatum Humb. & Bonpl. ex Flüggé	J	D	H	ADIC	H
Raddiella esenbeckii (Steud.) C.E. Calderón & Soderstr.	R;S	M	M	PT	NH
Schizachirium sanguineum (Retz.) Alston	F;H;S	D	H	PT	NH

S. tenerum Ness	S	D	AM	PT	NH
Sporobolus cubensis Hitchc.	R;S	D	AM	PT	NH
Thrasya trinitensis Mez	S	M	AM	PT	NH
Trachypogon spicatus (L. f.) Kuntze	F;H;J;L;M;R;S	M	H	PT	NH
Urochloa decumbens (Stapf) R.D. Webster	F;R	D	AM	PT	H
**POLYGALACEAE**					
Polygala adenophora DC.	S	D	H	ADIC	NH
P. appressa Benth.	H;L;M;R;S	M	H	ADIC	NH
P. glochidiata Kunth var. *glochidiata*	H;R;S	M	H	ADIC	NH
P. hygrophila Kunth	S	M	H	ADIC	NH
P. longicaulis Kunth	H;M;R;S	D	H	ADIC	NH
P. paniculata L.	F;S	D	H	ADIC	NH
P. timoutou Aubl.	R;S	D	H	ADIC	NH
P. violacea Aubl.	R	D	H	ADIC	NH
Securidaca paniculata Rich.	R	D	H	ADIC	H
**POLYGONACEAE**					
Coccoloba schomburgkii Meisn.	H;J;L;M	M	D[(hh)]	-	H
**PRIMULACEAE**					
Cybianthus crotonoides (R.M. Schomb. ex Mez) G. Agostini	S	M	D[(hh)]	-	H
C. duidae (Gleason & Moldenke) G. Agostini	L;M	M	D[(hh)]	-	H
C. quelchii (N.E. Br.) G. Agostini	J	M	D[(hh)]	-	H
Myrsine coriacea (Sw.) R. Br. ex Roem. & Schult.	J;R	D	D[(hh)**]	-	H
**PROTEACEAE**					
Panopsis sessilifolia Rich.	R	M	H	PT	H
Roupala minima Steyerm.	S	D	H	ADIC	H
R. montana Aubl.	M	M	H	PT	H
**RAPATEACEAE**					
Saxofrideria regalis R.H. Schomb.	J	M	H	ADIC	H
Stegolepis angustata Gleason	H;J;L;M	M	H	ADIC	H
S. ptaritepuiensis Steyerm.	H;J;L	M	H	ADIC	H
**RUBIACEAE**					
Borreria capitata (Ruiz & Pav.) DC.	J;R;S	D	H	ADIC	H
B. latifolia (Aubl.) K. Schum.	R	D	H	ADIC	H

B. vertillata (L.) G. Mey.	F	D	H	ADIC	H
Chalepophyllum guianense Hook f.	J;H;L;M	M	H(d)	ADIC	H
Coccocypselum hirsutum Bartl. ex DC.	R	D	H(d)	ADIC	H
Declieuxia fruticosa (Willd. ex Roem. & Schult.) Kuntze	F;J;M;R;S	M	H(d)	ADIC	H
Pagamea capitata Benth.	L;M	M	D(hh)	ADIC	H
Pagameopsis garryoides (Standl.) Steyerm.	J	M	H	ADIC	NH
Palicourea crocea (Sw.) Schult.	R	D	H(d)	ADIC	H
Perama dichotoma Poepp. var. *dichotoma*	H;L	D	H	ADIC	NH
P. galioides (Kunth) Poir.	F;H;J;L;M;R;S	D	H	ADIC	NH
Psychotria hoffmannseggiana (Shult.) Müll. Arg.	R	D	H(d)	ADIC	H
P. polycephala Benth.	L;M;R	D	H(d)	ADIC	H
Remijia densiflora Benth. ssp. *stenopetala* (Standl. & Steyerm.) Steyerm.	J;L	M	H(d)	ADIC	H
Sabicea velutina Benth.	J;L;M;R;S	D	H(d)	ADIC	H
Sipanea galioides Wernham	H;J;M;R;S	D	H(d)	ADIC	H
**SANTALACEAE**					
Dendrophthora elliptica (Gardner) Krug & Urb.	M;R	D	M	PT	H
Phoradendron crassifolium (Pohl ex DC.) Eichler	M	D	M	PT	H
P. piperoides (Kunth) Trel.	R	D	M	PT	H
P. semivenosum Rizzini	M	M	D(u)	-	-
P. strongyloclados Eichler	R	D	M	PT	H
Thesium tepuiense Steyerm.	H	M	H	ADIC	NH
**SAPINDACEAE**					
Matayba opaca *Radlk.*	J;L	M	AM	ADIC	H
**SAPOTACEAE**					
Elaeoluma schomburgkiana (Miq.) Baill.	L;M	M	GM	-	H
**SARRACENIACEAE**					
Heliamphora heterodoxa Steyerm.	J	M	H	PG	H
**SMILACACEAE**					
Smilax pittieriana Steyerm.	M	M	D(u)*	-	-
**SOLANACEAE**					
Melananthus ulei Carvalho	M	M	H	ADIC	NH
Solanum sect. Maurella	R	D	H	ADIC	H

Solanum campaniforme Roem. & Schult.	R;S	D	H	ADIC	H
Solanum stramoniifolium Jacq.	F;R	D	H	ADIC	H
**STYRACACEAE**					
Styrax wurdackiorum Steyerm.	J;M	M	H	ADIC	H
**VELLOZIACEAE**					
Vellozia tubiflora (A. Rich.) Kunth	L;S	M	H	PT	H
**VERBENACEAE**					
Lantana camara L.	F	D	H	ADIC	H
Stachytarpheta sprucei Moldenke	F	D	H	ADIC	H
**XYRIDACEAE**					
Abolboda acaulis Maguire	H;J;L;M	M	H	ADIC	H
A. macrostachya Spruce ex Malme var. *robustior* Steyerm.	H;J	M	H	ADIC	H
Orectanthe sceptrum (Oliv. ex Thurn) Maguire	H	M	H	ADIC	H
Xyris bicephala Gleason	H;J	M	H	ADIC	H
X. fallax Malme	H	M	H	ADIC	H
X. guianensis Steud.	H;L	M	H	ADIC	H
X. hymenachne Mart.	L	M	H	ADIC	H
X. involucrata Nees	H;J;L	M	H	ADIC	H
X. roraimae Malme	H	M	H	ADIC	H
X. setigera Oliv. ex Thurn	H;J;L;M;S	M	H	ADIC	H
X. seubertii Nilsson	L;M	M	H	ADIC	H
X. surinamensis Spreng.	L	M	H	ADIC	H
X. tenella Kunth	L	M	H	ADIC	H
X. uleana Malme	H;L	M	H	ADIC	H

[a] = Community: J = Shrubland (Jardin), L = Shrubland (Liworiwo), M = Shrubland (Mareman), R = Secondary bushland, H = Broad-leaved meadow, S = Savanna, B = Fallow

[b] = Habitat: M = Late seral or undisturbed area, D = disturbed area.

[c] = Sexuality: H = Hermaphrodite, M = Monoecy (only unisaxual flowers), AM = Andromonoecy, GM = Gynomonoecy, AGM = androgynomonoecious, D = Dioecy: ad = androdioecious, gd = gynodioecious, hh = hermaphrodite dimorphous, u = dioecious with unisexual flowers, AGD = androgynodioecious, d = dystylous flowers, * staminode in female flowers, **female rudiment in male flowers

[d] = TVSE = Temporal variation in sexual expression: AD = Adichogamy, PT = Protandry, PG = Protogyny.

[e] = Spatial variation of sexual organs: H = Herkogamy, NH = No Herkagamy.

Appendix 2. Results of experimental tests for 103 plant species from herbaceous-shrubby communities in the Gran Sabana Plateau.

FAMILY Species	Experimental test: Number of flower under experimental test				Number of fruit set				Ovule/ flower	Number of seed set			
	E	SSP	SP	CP	E	SSP	SP	CP	X[ϕ]	E	SSP	SP	CP
APOCYNACEAE													
Mandevilla benthamii	39	197	12	8	0	1	2	2	16.82	0	12	17	22
M. leptophylla	20	117	12	30	0	0	0	1	52.41	0	0	0	30
ASTERACEAE													
Chromolaena thurnii	210	420	120	120	0	251	25	32	1.00	0	251	25	32
Gongylolepis benthamiana	52	621	99	126	7	367	35	99	1.00	7	367	35	99
Stomatochaeta condensata	64	39	60	59	24	7	12	6	1.00	24	7	12	6
BIGNONIACEAE													
Digomphia laurifolia	7	18	17	15	0	0	7	3	23.76	0	0	55	32
BONNETIACEAE													
Bonnetia sessilis	65	172	55	47	0	1	18	11	354.30	0	69	2316	1294
BROMELIACEAE													
Brocchinia reducta	23	30	10	12	5	16	3	5	6.20	27	96	7	24
Catopsis berteroniana	26	234	28	22	0	55	10	13	31.90	0	655	180	282
Lindmania guianensis var. *vestita*	27	50	24	33	0	0	3	15	23.06	0	0	5	190
Tillandsia flexuosa	12	47	7	*	0	7	3	*	139.39	0	969	445	*
CALOPHYLLACEAE													
Mahurea exstipulata	12	62	*	*	0	0	*	*	777.30	0	0	*	*
CAMPANULACEAE													
Centropogon cornutus	17	22	6	*	1	1	2	*	2502.48	359	395	1208	*
CHRYSOBALANACEAE													
Hirtella scabra	20	102	69	49	0	1	4	4	2.00	0	1	4	4
CYPERACEAE													
Bulbostylis lanata	*	266	70	39	*	87	15	20	1.00	*	87	15	20
B. conifer	*	196	15	39	*	0	0	3	1.00	*	0	0	3
B. paradoxa	*	82	15	114	*	33	5	72	1.00	*	7	5	43
Hypolytrum pulchrum	*	75	150	12	*	0	0	2	1.00	*	0	0	2
Lagenocarpus rigidus	856	5152	35	50	0	38	11	15	1.00	0	213	11	15
Rhynchospora globosa	*	540	24	31	*	13	5	12	1.00	*	13	5	12
R. mexicana	19	28	*	39	0	0	*	4	1.00	0	0	*	4
R. pilosa	*	197	11	18	*	165	11	14	1.00	*	165	11	14
R. velutina	100	266	37	30	0	0	5	8	1.00	0	0	5	8
Scleria cyperina	68	135	100	63	1	13	13	20	1.00	1	13	13	20
CYRILLACEAE													
Cyrilla racemiflora	54	248	69	45	0	5	4	11	4.57	0	6	5	16
DROSERACEAE													
Drosera roraimae	24	50	*	*	0	25	*	*	125.54	0	1781	*	*
ERICACEAE													
Bejaria sprucei	53	136	23	38	2	18	2	22	325.86	139	603	42	2934
Notopora schomburgkii	32	134	19	25	0	2	0	13	133.89	0	16	0	1042
Vaccinium euryanthum	73	184	77	40	8	22	29	21	85.93	453	1596	1316	1406
EUPHRONIACEAE													
Euphronia guianensis	22	86	36	31	0	0	3	9	5.83	0	0	4	15

FABACEAE													
Calliandra resupina	22	108	13	44	0	0	1	1	5.77	0	0	1	1
Dicymbe fraterna	25	67	12	13	0	0	0	4	11.10	0	0	0	12
Dimorphandra macrostachya ssp. *macrostachya* **[2]**	100	1244	37	34	0	0	0	4	8.69	0	0	0	28
Taralea cordata	31	79	17	7^{β}	0	0	0	0	1.01	0	0	0	0
GENTIANACEAE													
Chelonanthus angustifolius	15	25	10	11	0	25	9	10	1278	0	22923	5807	9285
Irlbachia purpurascens	24	48	16	24	0	7	10	19	1914.63	0	1659	4021	4526
HYPERICACEAE													
Vismia guianensis	23	148	35	36	0	0	0	14	130.40	0	0	0	1199
HUMIRIACEAE													
Humiria balsamifera	61	268	101	81	0	0	4	15	8.65	0	0	6	29
Vantanea minor	34	268	35	33	0	1	0	4	9.15	0	2	0	7
IRIDACEAE													
Trimezia fosteriana	28	35	32	26	0	0	3	7	56.79	0	0	29	113
IXONANTHACEAE													
Ochthocosmus attenuatus	30	114	30	*	0	0	0	*	9.02	0	0	0	*
O. longipedicellatus	31	219	*	*	0	0	*	*	6.59	0	0	*	*
LOGANIACEAE													
Bonyunia minor	36	86	76	59	0	0	0	22	135.16	0	0	0	208
LORANTHACEAE													
Struthanthus gracilis	30	106	*	5^{β}	0	0	*	0	1.00	0	0	*	0
Tripodanthus acutifolius	46	87	49	147	0	0	4	41	1.00	0	0	4	41
MALPIGHIACEAE													
Byrsonima concinna	25	104	33	24	0	0	11	4	3.00	0	0	32	9
B. crassifolia (Shrublands)	10	85	19	62	0	0	6	10	3.00	0	0	8	17
B. crassifolia (Savanna)	19	206	22	36	0	0	0	16	3.00	0	0	0	29
B. verbascifolia	35	174	19	10	0	0	0	3	3.00	0	0	0	8
Tetrapterys pusilla	32	99	9	3^{β}	0	2	0	2	3.00	0	2	0	4
T. styloptera	18	42	10	15	0	0	0	3	3.00	0	0	0	5
MELASTOMATACEAE **[1]**													
Clidemia pustulata	55	57	19	13	6	15	3	9	206.30	1077	3153	417	1108
C. sericea	43	58	9	*	18	49	4	*	166.92	1122	2635	255	*
Comolia microphylla	36	49	33	20	2	0	14	13	43.76	34	0	305	417
Macairea parvifolia	68	242	59	34	4	9	4	0	49.07	164	281	133	0
Marcetia taxifolia	39	72	72	46	0	0	46	30	118.45	0	0	3918	1514
Meriania sclerophylla	33	104	25	26	0	0	8	8	180.42	0	0	1426	1559
M. urceolata	25	66	11	*	17	42	10	*	721.28	806	3228	895	*
Miconia alata	55	75	50	43	0	0	21	23	105.92	0	0	820	1341
M. albicans	89	86	*	*	29	51	*	*	36.66	514	1340	*	*
M. aplostachya	33	64	23	*	33	47	22	*	56.15	798	628	611	*
M. ciliata	41	94	18	12	3	3	0	5	104.39	214	237	0	79
M. ibaguensis	85	108	8	3^{β}	17	44	7	1	126.71	965	3914	372	76
M. phaeophylla	23	25	19	*	12	12	18	*	57.92	207	89	518	*
Microlicia benthamiana	47	22	9	11	6	5	6	1	66.94	248	349	211	36
Tibouchina fraterna	34	148	5^{β}	6^{β}	1	0	2	1	78.77	31	0	82	7
Tococa guianensis	30	32	17	17	0	0	0	11	187.34	0	0	0	1579
T. nitens	21	47	14	20	0	0	13	10	239.83	0	0	700	1005

MYRTACEAE														
Myrcia magnoliifolia		18	42	22	3^{β}	0	0	1	2	4.00	0	0	1	2
NARTHECIACEAE														
Nietneria paniculata		51	68	63	35	7	36	44	19	58.69	37	550	868	507
OCHNACEAE														
Ouratea gillyana		15	19	*	2^{β}	0	0	*	0	4.97	0	0	*	0
Poecilandra pumila		21	57	12	27	0	0	0	8	70.72	0	0	0	136
P. retusa		40	235	59	40	0	0	5	9	76.46	0	0	16	234
Sauvagesia guianensis		35	64	41	22	2	33	22	16	15.04	2	93	65	34
ORCHIDACEAE														
Cyrtopodium parviflorum		31	31	32	27	0	0	20	14	**	0	0	**	**
Epidendrum ibaguense		22	44	24	18	0	0	10	15	**	0	0	**	**
E. orchidiflorum		30	44	12	*	0	0	10	*	**	0	0	**	*
E. secundum		20	24	22	16	0	0	4	7	**	0	**	**	**
Eriopsis biloba		20	15	12	*	0	0	0	*	**	0	0	0	**
Koellensteinia eburnea		40	55	32	64	0	0	3	25	**	0	**	**	**
Sobralia elisabethae		15	20	16	16	0	0	15	11	**	0	**	**	**
PASSIFLORACEAE														
Passiflora sclerophylla		14	38	11	34	0	0	0	3	86.81	0	0	0	71
PENTAPHYLACACEAE														
Ternstroemia crassifolia		21	123	24	33	0	2	1	12	3.12	0	4	1	29
T. pungens		14	40	*	8	0	0	*	5	8.63	0	0	*	26
T. retusifolia		18	34	8	6	0	0	0	0	4.79	0	0	0	0
PHYLLANTHACEAE														
Phyllanthus major		*	56	8	26	*	0	3	0	6.00	*	0	17	0
POACEAE														
Axonopus anceps		24	224	40	61	0	0	2	5	1.00	0	0	2	5
Echinolaena inflexa		13	515	31	43	0	4	1	10	1.00	0	4	1	10
POLYGALACEAE														
Polygala longicaulis		*	67	12	19	*	55	11	9	2.00	*	109	20	16
P. paniculata		*	62	19	11	*	50	18	9	2.00	*	100	35	15
PROTEACEAE														
Roupala montana		13	77	40	22	0	0	0	0	2.00	0	0	0	0
RAPATEACEAE														
Stegolepis ptaritepuiensis		25	98	12	33	0	22	1	15	15.68	0	63	3	47
RUBIACEAE														
Chalepophyllum guianense	**[L]**	13	56	8	18(L*B)<br>21(L*L)	0	3	1	12(L*B)<br>2(L*L)	31.32	0	4	5	74(L*B)<br>3(L*L)
Chalepophyllum guianense	**[B]**	8	70	14	21(B*L)<br>20(B*B)	0	0	0	7(B*L)<br>2(B*B)	30.17	0	0	0	31(B*L)<br>5(L*L)
Palicourea crocea	**[L]**	27	68	47	23(L*B)<br>23(L*L)	0	1	1	1(L*B)<br>7(L*L)	1.93	0	2	1	1(L*B)<br>10(L*L)
Palicourea crocea	**[B]**	23	81	13	19(B*L)<br>19(B*B)	0	0	0	9(B*L)<br>0(B*B)	1.99	0	0	0	14(B*L)<br>0(B*B)
Sabicea velutina	**[L]**	10	28	11	21(L*B)<br>22(L*L)	0	0	0	12(L*B)<br>1(L*L)	179.19	0	0	0	1535(L*B)<br>264(L*L)
Sabicea velutina	**[B]**	6	22	11	17(B*L)<br>12(B*B)	0	0	0	8(B*L)<br>1(B*B)	144.89	0	0	0	661(B*L)<br>27(B*B)
Sipanea galioides	**[L]**	37	*	*	3(L*B)	0	*	*	3	61.13	0	*	*	152

SANTALACEAE													
Thesium tepuiense	19	115	19	20	7	45	9	11	3.91	7	45	9	11
SAPOTACEAE													
Elaeoluma schomburgkiana	22	51	28	37	0	0	0	6	3.00	0	0	0	8
SOLANACEAE													
Solanum (sec. *Maurella*)	20	52	24	22	0	8	7	13	60.40	0	51	257	659
STYRACACEAE													
Styrax wurdackiorum	19	38	32	18	0	3	8	6	16.51	0	3	6	6
XYRIDACEAE													
Abolboda macrostachya var. *robusta*	9	37	*	11	1	29	*	7	53.86	18	1305	*	235
Orectanthe sceptrum	24	156	*	*	15	109	*	*	151.13	1053	4639	*	*
Xyris bicephala	5$^{\beta}$	213	16	17	0	54	0	3	16.64	0	363	0	8

E = Emasculation; SSP = Spontaneous self-pollination; SP = Hand self-pollination; CP = Cross-pollinations; * = Test was not performed; ** = seed number were not counted; [L] = long style flower; [B] = Short Style flower. **[1]** Results from Hokche and Ramírez (2008); **[2]** Results from Ramírez and Briceño (2016); $^{\beta}$= Low number of flower, no considered for index calculation; $^{\phi}$ = Average value.

Appendix 3. Breeding system indexes and their qualitative categories.

FAMILIA / Species	Agamospermy index [AGI(sp)] Fruit level Index	Agamospermy index [AGI(sp)] Fruit level Category	Agamospermy index [AGI(sp)] Seed level Index	Agamospermy index [AGI(sp)] Seed level Category	Agamospermy index [AGI(cp)] Fruit level Index	Agamospermy index [AGI(cp)] Fruit level Category	Agamospermy index [AGI(cp)] Seed level Index	Agamospermy index [AGI(cp)] Seed level Category	Conclusion AGI[1]	Spontaneous self-pollination index (SSPI) Fruit level Index	SSPI Fruit level Category	SSPI Seed level Index	SSPI Seed level Category	Conclusion SSPI[2]	Self-fertility index (SFI) Fruit level Index	SFI Fruit level Category	SFI Seed level Index	SFI Seed level Category	Conclusion SFI[3]	Self-incompatibility index (ISI) Fruit level Index	ISI Fruit level Category	ISI Seed level Index	ISI Seed level Category	Conclusion ISI[4]	Composite Breeding System
**APOCYNACEAE**																									
Blepharodon pictum														NSSP*					X*						NSSP,X
B. ulei														NSSP*					X*						NSSP,X
Ditassa bolivarensis														NSSP*					X*						NSSP,X
D. tatei														NSSP*					X*						NSSP,X
Mandevilla benthamii	0.0000	NAG(sp)	0.0000	NAG(sp)	0.0000	NAG(cp)	0.0000	NAG(cp)	NAG	0.0305	NSSP	0.0430	PSSP	PSSP	0.0203	X	0.0222	PX	PX	0.6667	PSI	0.5152	PSI	PSI	NAG,NSSP,PX,PSI
M. gracilis	0.0000	NAG(sp)	0.0000	NAG(sp)	0.0000	NAG(cp)	0.0000	NAG(cp)	NAG	0.0000	NSSP	0.0000	NSSP	NSSP	0.0000	X	0.0000	X	X	0.0000	SI	0.0000	SI	SI	NAG,NSSP,X,SI
Metastelma hirtella														NSSP*					X*						NSSP,X
**ARACEAE**																									
Philodendron ptarianun var. *rugosum*														NSSP*					X*						NSSP,X
**ASTERACEAE**																									
Chromolaena thurnii	0.0000	NAG(sp)	0.0000	NAG(sp)	0.0000	NAG(cp)	0.0000	NAG(cp)	NAG	2.8686	PCASP	2.8686	PCASP	PCASP	2.2411	PE	2.2411	PE	PE	0.7813	PSI	0.7813	PSI	PSI	NAG,PCASP,PE,PSI
Gongylolepis benthamiana	0.3808	PAG(sp)	0.3808	PAG(sp)	0.1713	PAG(cp)	0.1713	PAG(cp)	PAG	1.6716	PCASP	1.6716	PCASP	PCASP	0.7522	PX	0.7522	PX	PX	0.4500	PSI	0.4500	PSI	PSI	PAG,PCASP,PX,PSI
Stomatochaeta condensata	1.8750	PCSM(sp)	1.8750	PCSM(sp)	3.6875	PCSM(sp)	3.6875	PCSM(sp)	PCSM	0.8974	PCASP	0.8974	PCASP	PCASP	1.7650	PE	1.7650	PE	PE	1.9667	P	1.9667	PCI	PCI	PCSM,PCASP,PE,PCI
**BIGNONIACEAE**																									
Digomphia laurifolia	0.0000	NAG(sp)	0.0000	NAG(sp)	0.0000	NAG(cp)	0.0000	NAG(cp)	NAG	0.0000	NSSP	0.0000	NSSP	NSSP	0.0000	X	0.0000	X	X	2.0588	PCI	1.5165	PCI	PCI	NAG,NSSP,X,PCI
**BONNETIACEAE**																									
Bonnetia sessilis	0.0000	NAG(sp)	0.0000	NAG(sp)	0.0000	NAG(cp)	0.0000	NAG(cp)	NAG	0.0178	NSSP	0.0095	PSSP	NSSP	0.0248	X	0.0146	PX	X	1.3983	PCI	1.5295	PCI	PCI	NAG,NSSP,X,PCI
**BROMELIACEAE**																									
Brocchinia reducta	0.7246	PAG(sp)	1.6770	PCSM(sp)	0.5217	PAG(cp)	0.5870	PAG(cp)	PAG	1.7778	PCASP	4.5714	PCASP	PCASP	1.2800	PE	1.6000	PE	PE	0.7200	PSI	0.3500	PSI	PSI	PAG,PCASP,PE,PSI
Catopsis berteroniana	0.0000	NAG(sp)	0.0000	NAG(sp)	0.0000	NAG(cp)	0.0000	NAG(cp)	NAG	0.6581	PSSP	0.4354	PSSP	PSSP	0.3978	PX	0.2184	PX	PX	0.6044	PSI	0.5015	PSI	PSI	NAG,PSSP,PX,PSI
Lindmania guianensis	0.0000	NAG(sp)	0.0000	NAG(sp)	0.0000	NAG(cp)	0.0000	NAG(cp)	NAG	0.0000	NSSP	0.0000	NSSP	NSSP	0.0000	X	0.0000	X	X	0.2750	PSI	0.0362	PSI	PSI	NAG,NSSP,X,PSI
Tillandsia flexuosa	0.0000	NAG(sp)	0.0000	NAG(sp)					NAG	0.3475	PSSP	0.3243	PSSP	PSSP											NAG,PSSP
**CAMPANULACEAE**																									
Centropogon cornutus	0.1765	PAG(sp)	0.1049	PAG(sp)					PAG	0.1364	PSSP	0.0892	PSSP	PSSP											PAG,PSSP
**CHRYSOBALANACEAE**																									
Hirtella scabra	0.0000	NAG(sp)	0.0000	NAG(sp)	0.0000	NAG(cp)	0.0000	NAG(cp)	NAG	0.1691	PSSP	0.1691	PSSP	PSSP	0.1201	PX	0.1201	PX	PX	0.7101	PSI	0.7101	PSI	PSI	NAG,PSSP,PX,PSI
**CYPERACEAE**																									
Bulbostylis conifera	0.0000	NAG(sp)	0.0000	NAG(sp)	0.0000	NAG(cp)	0.0000	NAG(cp)	NAG	0.0000	NSSP	0.0000	NSSP	NSSP	0.0000	X	0.0000	X	X	0.0000	SI	0.0000	SI	SI	NAG,NSSP,X,SI

B. lanata	0.0000	NAG(sp)	0.0000	NAG(sp)	0.0000	NAG(cp)	0.0000	NAG(cp)	NAG	1.5263	PCASP	1.5263	PCASP	PCASP	0.6378	PX	0.6378	PX	PX	0.4179	PSI	0.4179	PSI	PSI	NAG,PCASP,PX,PSI
B. paradoxa										0.2561	PSSP	0.2561	PSSP	PSSP	0.2263	PX	0.2263	PX	PX	0.8837	PSI	0.8837	PSI	PSI	PSSP,PX,PSI
Hypolytrum pulchrum	0.0000	NAG(sp)	0.0000	NAG(sp)	0.0000	NAG(cp)	0.0000	NAG(cp)	NAG	0.0000	NSSP	0.0000	NSSP	NSSP	0.0000	X	0.0000	X	X	0.0000	SI	0.0000	SI	SI	NAG,NSSP,X,SI
Lagenocarpus rigidus										0.0190	PSSP	0.0190	PSSP	PSSP	0.0232	PX	0.0232	PX	PX	1.2222	PCI	1.2222	PCI	PCI	PSSP,PX,PCI
Rhynchospora globosa										0.1100	PSSP	0.1100	PSSP	PSSP	0.0615	PX	0.0615	PX	PX	0.5384	PSI	0.5384	PSI	PSI	PSSP,PX,PSI
R. mexicana	0.0000	NAG(sp)	0.0000	NAG(sp)	0.0000	NAG(cp)	0.0000	NAG(cp)	NAG						0.0000	X	0.0000	X	X						NAG,X
R. pilosa										0.8376	PSSP	0.8376	PSSP	PSSP	1.0769	PE	1.0769	PE	PE	1.2857	PCI	1.2857	PCI	PCI	NAG,PSSP,PE,PCI
R. velutina	0.0000	NAG(sp)	0.0000	NAG(sp)	0.0000	NAG(cp)	0.0000	NAG(cp)	NAG	0.0000	NSSP	0.0000	NSSP	NSSP	0.0000	X	0.0000	X	X	0.5068	PSI	0.5068	PSI	PSI	NAG,NSSP,X,PSI
Scleria cyperina	0.0781	NAG(sp)	0.0781	NAG(sp)	0.0331	NAG(cp)	0.0331	NAG(cp)	NAG	0.7407	PSSP	0.7407	PSSP	PSSP	0.2167	PX	0.2167	PX	PX	0.4239	PSI	0.4239	PSI	PSI	NAG,PSSP,PX,PSI
**CYRILLACEAE**																									
Cyrilla racemiflora	0.0000	NAG(sp)	0.0000	NAG(sp)	0.0000	NAG(cp)	0.0000	NAG(cp)	NAG	0.3478	PSSP	0.3339	PSSP	PSSP	0.0825	PX	0.0680	PX	PX	0.2372	PSI	0.2038	PSI	PSI	NAG,PSSP,PX,PSI
**DROSERACEAE**																									
Drosera roraimae	0.0000	NAG(sp)	0.0000	NAG(sp)	0.0000	NAG(cp)	0.0000	NAG(cp)	NAG																NAG
**ERICACEAE**																									
Bejaria sprucei	0.4340	PAG(sp)	0.0652	NAG(sp)	1.4362	PCSM(cp)	0.0340	NAG(cp)	NAG	1.5221	PCASP	2.4280	PCASP	PCASP	0.2286	PX	0.0574	PX	PX	0.1502	SI	0.0237	PSI	PSI	PAG,PCASP,PX,PSI
Notopora schomburgkii	0.0000	NAG(sp)	0.0000	NAG(sp)	0.0000	NAG(cp)	0.0000	NAG(cp)	NAG	0.0000	NSSP	0.0000	NSSP	NSSP	0.0287	X	0.0029	X	X	0.0000	SI	0.0000	SI	SI	NAG,NSSP,X,SI
Vaccinium euryanthum	0.2910	PAG(sp)	0.3631	PAG(sp)	0.2087	PAG(cp)	0.1765	PAG(cp)	PAG	0.3175	PSSP	0.5075	PSSP	PSSP	0.2277	PX	0.2468	PX	PX	0.7174	PSI	0.4862	PSI	PSI	PAG,PSSP,PX,PSI
**EUPHRONIACEAE**																									
Euphronia guianensis	0.0000	NAG(sp)	0.0000	NAG(sp)	0.0000	NAG(cp)	0.0000	NAG(cp)	NAG	0.0000	NSSP	0.0000	NSSP	NSSP	0.0000	X	0.0000	X	X	0.2870	PSI	0.2296	PSI	PSI	NAG,NSSP,X,PSI
**FABACEAE**																									
Calliandra pakaraimensis	0.0000	NAG(sp)	0.0000	NAG(sp)	0.0000	NAG(cp)	0.0000	NAG(cp)	NAG	0.0000	NSSP	0.0000	NSSP	NSSP	0.0000	X	0.0000	X	X	3.3846	PCI	3.3846	PCI	PCI	NAG,NSSP,X,P
Dicymbe fraterna	0.0000	NAG(sp)	0.0000	NAG(sp)	0.0000	NAG(cp)	0.0000	NAG(cp)	NAG	0.0000	NSSP	0.0000	NSSP	NSSP	0.0000	X	0.0000	X	X	0.0000	SI	0.0000	SI	SI	NAG,NSSP,X,SI
Dimorphandra macrostachya ssp. *macrostachya*	0.0000	NAG(sp)	0.0000	NAG(sp)	0.0000	NAG(cp)	0.0000	NAG(cp)	NAG	0.0000	NSSP	0.0000	NSSP	NSSP	0.0000	X	0.0000	X	X	0.0000	SI	0.0000	SI	SI	NAG,NSSP,X,SI
Taralea cordata	0.0000	NAG(sp)	0.0000	NAG(sp)					NAG	0.0000	NSSP	0.0000	NSSP	NSSP					X						NAG,NSSP
**GENTIANACEAE**																									
Chelonanthus angustifolius	0.0000	NAG(sp)	0.0000	NAG(sp)	0.0000	NAG(cp)	0.0000	NAG(cp)	NAG	1.1111	PCASP	1.5790	PCASP	PCASP	1.1000	PE	1.0863	PE	PE	0.9900	SC	0.6880	PSI	PSI	NAG,PCASP,PE,PSI
C. purpurascens	0.0000	NAG(sp)	0.0000	NAG(sp)	0.0000	NAG(cp)	0.0000	NAG(cp)	NAG	0.2333	PSSP	0.1375	PSSP	PSSP	0.1842	PX	0.1833	PX	PX	0.7895	PSI	1.3326	PCI	PCI	NAG,PSSP,PX,PCI
**HUMIRIACEAE**																									
Humiria balsamifera	0.0000	NAG(sp)	0.0000	NAG(sp)	0.0000	NAG(cp)	0.0000	NAG(cp)	NAG	0.0000	NSSP	0.0000	NSSP	NSSP	0.0000	X	0.0000	X	X	0.2139	PSI	0.1659	PSI	PSI	NAG,NSSP,X,PSI
Vantanea minor	0.0000	NAG(sp)	0.0000	NAG(sp)	0.0000	NAG(cp)	0.0000	NAG(cp)	NAG	0.0000	NSSP	0.0000	NSSP	NSSP	0.0616	X	0.0352	PX	X	0.0000	SI	0.0000	SI	SI	NAG,NSSP,X,SI
**HYPERICACEAE**																									
Vismia guianensis	0.0000	NAG(sp)	0.0000	NAG(sp)	0.0000	NAG(cp)	0.0000	NAG(cp)	NAG	0.0000	NSSP	0.0000	NSSP	NSSP	0.0000	X	0.0000	X	X	0.0000	SI	0.0000	SI	SI	NAG,NSSP,X,SI
**IRIDACEAE**																									

Trimezia fosteriana	0.0000	NAG(sp)	0.0000	NAG(sp)	0.0000	NAG(cp)	0.0000	NAG(cp)	NAG	0.0000	NSSP	0.0000	NSSP	NSSP	0.0000	X	0.0000	X	X	0.3482	PSI	0.2085	PSI	PSI	NAG,NSSP,X,PSI
**IXONANTHACEAE**																									
Ochthocosmus attenuatus	0.0000	NAG(sp)	0.0000	NAG(sp)	0.0000	NAG(cp)	0.0000	NAG(cp)	NAG	0.0000	NSSP	0.0000	NSSP	NSSP	0.0000	X	0.0000	X	X	0.0000	SI	0.0000	SI	SI	NAG,NSSP,X,SI
O. longipedicellatus	0.0000	NAG(sp)	0.0000	NAG(sp)	0.0000	NAG(cp)	0.0000	NAG(cp)	NAG	0.0000	NSSP	0.0000	NSSP	NSSP											NAG,NSSP
**LOGANIACEAE**																									
Bonyunia minor	0.0000	NAG(sp)	0.0000	NAG(sp)	0.0000	NAG(cp)	0.0000	NAG(cp)	NAG	0.0000	NSSP	0.0000	NSSP		0.0000	X	0.0000	X	X	0.0000	SI	0.0000	SI	SI	NAG,NSSP,X,SI
**LORANTHACEAE**																									
Struthanthus gracilis	0.0000	NAG(sp)	0.0000	NAG(sp)	0.0000	NAG(cp)	0.0000	NAG(cp)	NAG	0.0000	NSSP	0.0000	NSSP	NSSP	0.0000	X	0.0000	X	X						NAG,NSSP,X
Tripodanthus acutifolius	0.0000	NAG(sp)	0.0000	NAG(sp)	0.0000	NAG(cp)	0.0000	NAG(cp)	NAG	0.0000	NSSP	0.0000	NSSP	NSSP	0.0000	X	0.0000	X	X	0.2927	PSI	0.2927	PSI	PSI	NAG,NSSP,X,PSI
**MALPIGHIACEAE**																									
Byrsonima concinna	0.0000	NAG(sp)	0.0000	NAG(sp)	0.0000	NAG(cp)	0.0000	NAG(cp)	NAG	0.0000	NSSP	0.0000	NSSP	NSSP	0.0000	X	0.0000	X	X	2.0050	PCI	2.5859	PCI	PCI	NAG,NSSP,X,PCI
B. crassifolia (Shrubland)	0.0000	NAG(sp)	0.0000	NAG(sp)	0.0000	NAG(cp)	0.0000	NAG(cp)	NAG	0.0000	NSSP	0.0000	NSSP	NSSP	0.0000	X	0.0000	X	X	1.9579	PCI	1.5356	PCI	PCI	NAG,NSSP,X,PCI
B. crassifolia (Savanna)	0.0000	NAG(sp)	0.0000	NAG(sp)	0.0000	NAG(cp)	0.0000	NAG(cp)	NAG	0.0000	NSSP	0.0000	NSSP	NSSP	0.0000	X	0.0000	X	X	0.0000	SI	0.0000	SI	SI	NAG,NSSP,X,SI
B. verbascifolia	0.0000	NAG(sp)	0.0000	NAG(sp)	0.0000	NAG(cp)	0.0000	NAG(cp)	NAG	0.0000	NSSP	0.0000	NSSP	NSSP	0.0000	X	0.0000	X	X	0.0000	SI	0.0000	SI	SI	NAG,NSSP,X,SI
Tetrapterys styloptera	0.0000	NAG(sp)	0.0000	NAG(sp)	0.0000	NAG(cp)	0.0000	NAG(cp)	NAG	0.0000	NSSP	0.0000	NSSP	NSSP	0.0000	X	0.0000	X	X	0.0000	SI	0.0000	SI	SI	NAG,NSSP,X,SI
T. pusilla	0.0000	NAG(sp)	0.0000	NAG(sp)	0.0000	NAG(cp)	0.0000	NAG(cp)	NAG	0.0000	NSSP	0.0000	NSSP	NSSP	0.0303	X	0.0152	X	X	0.0000	SI	0.0000	SI	SI	NAG,NSSP,X.SI
**MELASTOMATACEAE**																									
Clidemia pustulata	0.6909	PAG(sp)	0.8922	PAG(sp)	0.1576	PAG(cp)	0.2298	PAG(cp)	PAG	1.6667	PCASP	2.5204	PCASP	PCASP	0.3801	PX	0.6490	PX	PX	0.2281	PSI	0.2575	PSI	PSI	PAG,PCASP,PX,PSI
C. sericea	0.9419	PAG(sp)	0.9209	PAG(sp)					PAG	1.8686	PCASP	1.5763	PCASP	PCASP											PAG,PCASP
Comolia microphylla	0.1310	NAG(sp)	0.1022	PAG(sp)	0.0855	NAG(cp)	0.0453	PAG(cp)	NAG	0.0000	NSSP	0.0000	NSSP	NSSP	0.0000	X	0.0000	X	X	0.6527	PSI	0.4433	PSI	PSI	NAG,NSSP,X,PSI
Macairea parvifolia	0.8676	PAG(sp)	1.0699	PCSM(sp)					PAG	0.5486	PSSP	0.5151	PSSP	PSSP											PAG,PSSP
Marcetia taxifolia	0.0000	NAG(sp)	0.0000	NAG(sp)	0.0000	NAG(cp)	0.0000	NAG(cp)	NAG	0.0000	NSSP	0.0000	NSSP	NSSP	0.0000	X	0.0000	X	X	0.9796	SC	1.6533	PCI	PCI	NAG,NSSP,X,PCI
Meriania sclerophylla	0.0000	NAG(sp)	0.0000	NAG(sp)	0.0000	NAG(cp)	0.0000	NAG(cp)	NAG	0.0000	NSSP	0.0000	NSSP	NSSP	0.0000	X	0.0000	X	X	1.0400	SC	0.9513	PSI	PSI	NAG,NSSP,X,PSI
M. urceolata	0.6679	PAG(sp)	0.3538	PAG(sp)					PAG	0.7000	PSSP	0.6011	PSSP	PSSP											PAG,PSSP
Miconia alata	0.0000	NAG(sp)	0.0000	NAG(sp)	0.0000	NAG(cp)	0.0000	NAG(cp)	NAG	0.0000	NSSP	0.0000	NSSP	NSSP	0.0000	X	0.0000	X	X	0.7852	PSI	0.5259	PSI	PSI	NAG,NSSP,X,PSI
M. aplostachya	1.0455	PCSM(sp)	0.9103	PAG(sp)					PAG	0.7678	PSSP	0.3694	PSSP	PSSP											PAS,PSSP
M. ciliata	0.0000	NAG(sp)	0.0000	NAG(sp)	0.1756	NAG(cp)	0.7928	PAG(cp)	PAG	0.0000	NSSP	0.0000	NSSP	NSSP	0.0766	X	0.3830	PX	X	0.0000	SI	0.0000	SI	SI	PAG,NSSP,X,SI
M. ibaguensis	0.2286	PAG(sp)	0.2441	PAG(sp)			0.4481	PAG(cp)	PAG	0.4656	PSSP	0.7794	PSSP	PSSP			1.4306	PE	PE			1.8355	PCI	PCI	PAG,PSSP,PE,PCI
M. phaeophylla	0.5507	PAG(sp)	0.3301	PAG(sp)					PAG	0.5067	PSSP	0.1306	PSSP	PSSP											PAG,PSSP
Microlicia benthamiana	0.1702	PAG(sp)	0.2001	PAG(sp)	1.4043	PCSM(cp)	1.6123	PCSM(cp)	PCSM	0.3030	PSSP	0.6015	PSSP	PSSP	2.5000	PE	4.8472	PE	PE	8.2500	CI	8.0590	PCI	PCI	PCSM,PSSP,PE,PCI
Tibouchina fraterna	0.0735	NAG(sp)	0.0556	PAG(sp)					PAG	0.0000	NSSP	0.0000	NSSP	NSSP			0.0000	X	X			14.0571	PCI	PCI	PAG,NSSP,X,PCI
Tococa guianensis	0.0000	NAG(sp)	0.0000	NAG(sp)	0.0000	NAG(cp)	0.0000	NAG(cp)	NAG	0.0000	NSSP	0.0000	NSSP	NSSP	0.0000	X	0.0000	X	X	0.0000	SI	0.0000	SI	SI	NAG,NSSP,X,SI
T. nitens	0.0000	NAG(sp)	0.0000	NAG(sp)	0.0000	NAG(cp)	0.0000	NAG(cp)	NAG	0.0000	NSSP	0.0000	NSSP	NSSP	0.0000	X	0.0000	X	X	1.8571	PCI	0.9950	PSI	PSI	NAG,NSSP,X,PSI
**MYRTACEAE**																									

Myrcia magnoliifolia	0.0000	NAG(sp)	0.0000	NAG(sp)	0.0000	NAG(cp)	0.0000	NAG(cp)	NAG	0.0000	NSSP	0.0000	NSSP	NSSP	0.0000	X	0.0000	X	X	0.0682	SI	0.0682	SI	SI	NAG,NSSP,X,SI
**NARTHECIACEAE**																									
Nietneria paniculata	0.1965	PAG(sp)	0.0527	PAG	0.2528	PAG(cp)	0.0501	PAG	PAG	0.7580	PSSP	0.5870	PSSP	PSSP	0.9752	A	0.5584	PX	PX	1.2865	PCI	0.9511	PSI	PSI	PAG,PSSP,PX,PSI
**OCHNACEAE**																									
Poecilandra pumila	0.0000	NAG(sp)	0.0000	NAG(sp)	0.0000	NAG(cp)	0.0000	NAG(cp)	NAG	0.0000	NSSP	0.0000	NSSP	NSSP	0.0000	X	0.0000	X	X	0.0000	SI	0.0000	SI	SI	NAG,NSSP,X,SI
P. retusa	0.0000	NAG(sp)	0.0000	NAG(sp)	0.0000	NAG(cp)	0.0000	NAG(cp)	NAG	0.0000	NSSP	0.0000	NSSP	NSSP	0.0000	X	0.0000	X	X	0.3766	PSI	0.0464	PSI	PSI	NAG,NSSP,X,PSI
Sauvagesia guianensis	0.1065	NAG(sp)	0.0360	NAG(sp)	0.0786	NAG(cp)	0.0370	NAG(cp)	NAG	0.9609	PSSP	0.9166	PSSP	PSSP	0.7090	PX	0.9403	PX	PX	0.7378	PSI	1.0258	SC	PSI	NAG,PSSP,PX,PSI
**ORCHIDACEAE**																									
Cyrtopodium parviflorum	0.0000	NAG(sp)			0.0000	NAG(cp)			NAG	0.0000	NSSP			NSSP	0.0000	X			X	1.2054	PCI			PCI	NAG,NSSP,X,PCI
Echinosepala arenicola														NSSP*					X*						NSSP,X
Epidendrum dendrobioides														NSSP*					X*						NSSP,X
E. ibaguense	0.0000	NAG(sp)			0.0000	NAG(cp)			NAG	0.0000	NSSP			NSSP	0.0000	X			X	0.5000	PSI			PSI	NAG,NSSP,X,PSI
E. orchidiflorum	0.0000	NAG(sp)								0.0000	NSSP			NSSP					X*						NAG,NSSP
E. secundum	0.0000	NAG(sp)			0.0000	NAG(cp)			NAG	0.0000	NSSP			NSSP	0.0000	X			X	0.4156	PSI			PSI	NAG,NSSP,X,PSI
E. tumuc-humaciense														NSSP*					X*						NSSP,X
Epistephium duckei														NSSP*					X*						NSSP,X
E. subrepens														NSSP*					X*						NSSP,X
Eriopsis biloba	0.0000	NAG(sp)			0.0000	NAG(cp)				0.0000	NSSP			NSSP	0.0000	X			X						NAG,NSSP,X
Koellensteinia tricolor	0.0000	NAG(sp)			0.0000	NAG(cp)			NAG	0.0000	NSSP			NSSP	0.0000	X			X	0.2400	PSI			PSI	NAG,NSSP,X,PSI
Maxillaria auyantepuiensis														NSSP*					X*						NSSP,X
Scaphyglottis bidentata														NSSP*					X*						NSSP,X
Sobralia elisabethae	0.0000	NAG(sp)			0.0000	NAG(cp)			NAG	0.0000	NSSP			NSSP	0.0000	X			X	1.3636	PCI			PCI	NAG,NSSP,X,PCI
**PASSIFLORACEAE**																									
Passiflora sclerophylla	0.0000	NAG(sp)	0.0000	NAG(sp)	0.0000	NAG(cp)	0.0000	NAG(cp)	NAG	0.0000	NSSP	0.0000	NSSP	NSSP	0.0000	X	0.0000	X	X	0.0000	SI	0.0000	SI	SI	NAG,NSSP,X,SI
**PENTAPHYLACACEAE**																									
Ternstroemia crassifolia	0.0000	NAG(sp)	0.0000	NAG(sp)	0.0000	NAG(cp)	0.0000	NAG(cp)	NAG	0.3902	PSSP	0.7805	PSSP	PSSP	0.0447	X	0.0370	PX	PX	0.1146	SI	0.0474	SI	SI	NAG,PSSP,X,SI
T. pungens						NAG(cp)	0.0000	NAG(cp)	NAG						0.0000	X	0.0000	X	X						NAG,X
T. retusifolia	0.0000	NAG(sp)	0.0000	NAG(sp)	0.0000				NAG			0.0000	NSSP	NSSP					X						NAG,NSSP
**PHYLLANTHACEAE**																									
Phyllanthus majus	0.0000	NAG(sp)	0.0000	NAG(sp)	0.0000	NAG(cp)	0.0000	NAG(cp)	NAG	0.0000	NSSP	0.0000	NSSP	NSSP	0.0000	X	0.0000	X	X	∞	CI	∞	CI	CI	NAG,NSSP,X,CI
P. stipulatus														NSSP*					X						NSSP,X
**POACEAE**																									
Axonopus anceps	0.0000	NAG(sp)	0.0000	NAG(sp)	0.0000	NAG(cp)	0.0000	NAG(cp)	NAG	0.0000	NSSP	0.0000	NSSP	NSSP	0.0000	X	0.0000	X	X	0.6100	PSI	0.6100	PSI	PSI	NAG,NSSP,X,PSI
Echinolaena inflexa	0.0000	NAG(sp)	0.0000	NAG(sp)	0.0000	NAG(cp)	0.0000	NAG(cp)	NAG	0.1299	PSSP	0.1299	PSSP	PSSP	0.0279	X	0.0279	X	X	0.2150	PSI	0.2150	PSI	PSI	NAG,PSSP,X,PSI

**POLYGALACEAE**																									
Polygala longicaulis										0.8955	PSSP	0.9761	PSSP	PSSP	1.7330	PE	1.9319	PE	PE	1.9352	PCI	1.9792	PCI	PCI	PSSP,PE,PCI
P. paniculata										0.8513	PSSP	0.8756	PSSP	PSSP	0.9857	A	1.1828	PE	PE	1.1579	PCI	1.3509	PCI	PCI	PSSP,PE,PCI
**PROTEACEAE**																									
Roupala montana	0.0000	NAG(sp)	0.0000	NAG(sp)					NAG	0.0000	NSSP	0.0000	NSSP	NSSP					X						NAG,NSSP
**RAPATEACEAE**																									
Stegolepis ptaritepuiensis	0.0000	NAG(sp)	0.0000	NAG(sp)	0.0000	NAG(cp)	0.0000	NAG(cp)	NAG	2.7500	PCASP	2.6250	PCASP	PCASP	0.5042	PX	0.4608	PX	PX	0.1833	PSI	0.1755	PSI	PSI	NAG,PCASP,PX,PSI
**RUBIACEAE**																									
Chalepophyllum guianense (B)	0.0000	NAG(sp)	0.0000	NAG(sp)	0.0000	NAG(cp)	0.0000	NAG(cp)	NAG	0.0000	NSSP	0.0000	NSSP	NSSP	0.0000	X	0.0000	X	X	0.0000	SI	0.0000	SI	SI	NAG,NSSP,X,SI
Chalepophyllum guianense (L)	0.0000	NAG(sp)	0.0000	NAG(sp)	0.0000	NAG(cp)	0.0000	NAG(cp)	NAG	0.4286	PSSP	0.1143	PSSP	PSSP	0.0938	X	0.0203	PX	X	0.2188	PSI	0.1774	PSI	PSI	NAG,PSSP,X,PSI
Palicourea crocea (L)	0.0000	NAG(sp)	0.0000	NAG(sp)	0.0000	NAG(cp)	0.0000	NAG(cp)	NAG	0.6912	PSSP	1.3824	PCASP	PCASP	0.0483	X	0.0676	X	X	0.0699	SI	0.0489	SI	SI	NAG,PCASP,X,SI
Palicourea crocea (B)	0.0000	NAG(sp)	0.0000	NAG(sp)	0.0000	NAG(cp)	0.0000	NAG(cp)	NAG	0.0000	NSSP	0.0000	NSSP	NSSP	0.0000	X	0.0000	X	X	0.0000	SI	0.0000	SI	SI	NAG,NSSP,X,SI
Sabicea velutina (L)	0.0000	NAG(sp)	0.0000	NAG(sp)	0.0000	NAG(cp)	0.0000	NAG(cp)	NAG	0.0000	NSSP	0.0000	NSSP	NSSP	0.0000	X	0.0000	X	X	0.0000	SI	0.0000	SI	SI	NAG,NSSP,X,SI
Sabicea velutina (B)	0.0000	NAG(sp)	0.0000	NAG(sp)	0.0000	NAG(cp)	0.0000	NAG(cp)	NAG	0.0000	NSSP	0.0000	NSSP	NSSP	0.0000	X	0.0000	X	X	0.0000	SI	0.0000	SI	SI	NAG,NSSP,X,SI
Sipanea galioides (L)						NAG(cp)	0.0000	NAG(cp)	NAG						0.0000	X	0.0000	X	X						NAG,X
**SANTALACEAE**																									
Dendrophthora elliptica														NSSP*					X						NSSP,X
Phoradendron crassifolium														NSSP*					X						NSSP,X
P. piperoides														NSSP*					X						NSSP,X
P. strongyloclados														NSSP*					X*						NSSP,X
Thesium tepuiense	0.7778	PAG(sp)	0.7778	PAG(sp)	0.6699	PAG(sp)	0.6699	PAG(sp)	PAG	0.8261	PSSP	0.8261	PSSP	PSSP	0.7115	PX	0.7115	PX	PX	0.8612	PSI	0.8612	PSI	PSI	PAG,PSSP,PX,PSI
**SAPOTACEAE**																									
Elaeoluma schomburgkiana	0.0000	NAG(sp)	0.0000	NAG(sp)	0.0000	NAG(cp)	0.0000	NAG(cp)	NAG	0.0000	NSSP	0.0000	NSSP	NSSP	0.0000	X	0.0000	X	X	0.0000	SI	0.0000	SI	SI	NAG,NSSP,X,SI
**SOLANACEAE**																									
Solanum sect. Maurella	0.0000	NAG(sp)	0.0000	NAG(sp)	0.0000	NAG(cp)	0.0000	NAG(cp)	NAG	0.5275	PSSP	0.0916	PSSP	PSSP	0.2604	PX	0.0327	PX	PX	0.4936	PSI	0.3575	PSI	PSI	NAG,PSSP,PX,PSI
**STYRACACEAE**																									
Styrax wurdackiorum	0.0000	NAG(sp)	0.0000	NAG(sp)	0.0000	NAG(cp)	0.0000	NAG(cp)	NAG	0.4211	PSSP	0.4211	PSSP	PSSP	0.2368	PX	0.2368	PX	PX	0.5625	PSI	0.5625	PSI	PSI	NAG,PSSP,PX,PSI
**XYRIDACEAE**																									
Abolboda macrostachya var. *robusta*					0.1746	NAG(cp)	0.0936	PAG(cp)	NAG						1.2317	PE	1.6509	PE	PE						NAG,PE
Xyris bicephala	0.0000	NAG(sp)	0.0000	NAG(sp)	0.0000	NAG(cp)	0.0000	NAG(cp)	NAG	∞	OSSP	∞	OSSP	OSSP	1.4366	PE	3.6215	PE	PE	0.0000	SI	0.0000	SI	SI	NAG,OSSP,PE,SI

* Category suggested by:1- pollen grouped into masses, pollinia (Asclepiadoideae in the Apocynaceae) and (Orchidoideae and Epidendroideae in the Orchidaceae), and 2- monoecious-hercogamous-dichogamous species (Philodendron, Phyllanthus, Phoradendron). [AGI(sp)] = Agamospermy index determined as dividing the results obtained from the agamospermy test by the results obtained from the self-pollination test. [AGI(cp)] = Agamospermy index determined as dividing the results obtained from the agamospermy test by the results obtained from the cross-pollination. [1] = NAG (Non-agamospermous); PAG (Partially agamospermous); PCSM (Partially constrained sexual mating). [2] = NSSP (Non-spontaneous self-pollinated); PSSP (Partially spontaneous self-pollinated); PCASP (Partially constrained assisted self-pollination). [3] X (Xenogamous); PX (Partially xenogamous); PE (Partially endogamous). [4] = SI (Self-incompatible); PSI (Partially self-incompatible); SC (Self-compatible); PCI (Partially cross-incompatible); CI (Cross-incompatible). ∞ = indicate values divide by cero (1/BSI ~ 0)
